# Interrelated involvement of the endocannabinoid/endovanilloid (TRPV1) systems and epigenetic processes in anxiety‐ and working memory impairment‐related behavioural effects of nicotine as a stressor

**DOI:** 10.1111/adb.13421

**Published:** 2024-07-04

**Authors:** Tamaki Hayase

**Affiliations:** ^1^ Department of Legal Medicine Kyoto University Kyoto Japan

**Keywords:** anxiety/working memory, endocannabinoid, endovanilloid (TRPV1), epigenetic histone acetylation, nicotine, stress

## Abstract

The addictive use of nicotine contained in tobacco is associated with stressor‐like emotional and cognitive effects such as anxiety and working memory impairment, and the involvement of epigenetic mechanisms such as histone acetylation has recently been reported. Although the precise nature of behavioural plasticity remains unclear, both anxiogenic‐ and working memory impairment‐like effects were observed in the present experimental model of mice treated with repeated subcutaneous nicotine and/or immobilization stress, and these effects were commonly attenuated by the histone deacetylase (HDAC) inhibitors that induce histone acetylation. Such HDAC inhibitor‐induced resilience was mimicked by ligands for the endocannabinoid (ECB) system, a neurotransmitter system that is closely associated with nicotine‐induced addiction‐related behaviours: the anxiogenic‐like effects were mitigated by the cannabinoid type 1 (CB1) agonist arachidonylcyclopropylamide (ACPA), whereas the working memory impairment‐like effects were mitigated by the CB1 antagonist SR 141716A. Moreover, the effects of the HDAC inhibitors were also mimicked by ligands for the endovanilloid (transient receptor potential vanilloid 1 [TRPV1]) system, a system that shares common characteristics with the ECB system: the anxiogenic‐like effects were mitigated by the TRPV1 antagonist capsazepine, whereas the working memory impairment‐like effects were mitigated by the TRPV1 agonist olvanil. Notably, the HDAC inhibitor‐induced anxiolytic‐like effects were attenuated by SR 141716A, which were further counteracted by capsazepine, whereas the working memory improvement‐like effects were attenuated by capsazepine, which were further counteracted by SR 141716A. These results suggest the contribution of interrelated control of the ECB/TRPV1 systems and epigenetic processes such as histone acetylation to novel therapeutic approaches.

## Graphical abstract

Both anxiogenic‐ and working memory impairment‐like effects were observed in mice treated with repeated nicotine (NC) and/or immobilization stress (IM), and these effects were attenuated by the histone deacetylase (HDAC) inhibitor SB or VA. Notably, the HDAC inhibitor‐induced anxiolytic‐like effects were attenuated by the cannabinoid type 1 antagonist SR, which were further counteracted by the transient receptor potential vanilloid 1 antagonist CZ, whereas the working memory improvement‐like effects were attenuated by CZ, which were further counteracted by SR.

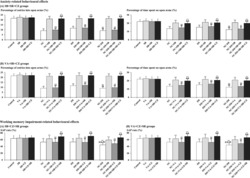

## 1 INTRODUCTION

Nicotine (NC) is known to be a highly addictive ingredient of tobacco that diminishes autonomy over smoking and affects, possibly exacerbates, the severity of coronavirus disease 2019 (COVID‐19) by modulating the target nicotinic acetylcholine receptors (nAChRs) and relevant molecular systems (e.g., angiotensin‐converting enzyme 2‐related system).^1^, ^2^, ^3^ Chronic NC exposure results in increased emotional symptoms such as anxiety, and concurrent cognitive deficits such as impaired working memory have often been reported.^4^ Like NC consumed by cigarette smokers, various stressors induce anxiety as a defensive behavioural response, which is accompanied by cognitive problems.^5^, ^6^ Along with “stressor‐like” behavioural effects, the dysregulated stress response in the brain has also been reported as NC‐induced plasticity.^7^, ^8^ Moreover, in some stressor‐exposed smokers, pronounced exacerbations of both emotional symptoms and cognitive deficits have been observed.^9^, ^10^ On the other hand, anxiolytic, memory‐improving and stress‐relieving effects have also been reported depending on the smoking‐related condition.^11^, ^12^ With respect to such behavioural heterogeneity, the interrelated contributions of multiple mechanisms including the characteristic bimodal involvement of nAChRs, the direct molecular targets of NC, along with the antistress effects of the main NC metabolite cotinine have been suggested,^13^, ^14^, ^15^ but the details have not been elucidated.

Epigenetic mechanisms, which regulate chromatin structure and gene expression, are associated with synaptic/circuitry function related to drug addiction and other psychiatric conditions.^16^, ^17^ Recently, growing evidence has indicated the involvement of impaired epigenetic processes in stress‐related behavioural plasticity.^18^, ^19^, ^20^ In particular, an increasing number of studies have identified the prominent contribution of attenuated histone acetylation to stressor‐induced behavioural impairments, based on its regulatory influence on the stress‐related neurotransmitter systems and the other epigenetic processes such as DNA methylation.^21^, ^22^, ^23^ Although epigenetic processes in the stress‐related behavioural effects of NC remain insufficiently explored, a pivotal integrative role of histone acetylation in the brain has been suggested for NC‐related behavioural plasticity.^24^, ^25^

Among the neurotransmitter systems implicated in NC‐induced behavioural and synaptic plasticity, the endocannabinoid (ECB) system has been considered to play important roles in the manifestation of addictive behaviours, based on the distributionally and functionally extensive crosstalk with the target neurotransmitter systems of NC (e.g., nicotinic cholinergic system).^26^, ^27^, ^28^ Increasing attention has also been focused on the involvement of the ECB system in stress‐related emotional and cognitive responses.^29^, ^30^ On the ECB system‐related behavioural plasticity, the crucial influence of the endovanilloid (transient receptor potential vanilloid 1 [TRPV1]) system, which was originally characterized as a molecular integrator of physical and chemical stimuli,^31^, ^32^ has been suggested, based on the neuroanatomical/functional similarity and close relationship between these systems, along with mutual regulation by dual‐acting endogenous ligands.^33^, ^34^, ^35^, ^36^, ^37^ However, little is known about the epigenetic mechanisms associated with the ECB‐TRPV1 crosstalk. In the present experimental study, the interacting influence of ECB/TRPV1 system‐related ligands and histone deacetylase (HDAC) inhibitors, which induce histone acetylation, on the NC‐ and/or stressor‐induced anxiety‐ and working memory‐related behaviours, is evaluated, and therapeutic importance of the mutual control of the ECB/TRPV1 systems and epigenetic processes is discussed.

## 2 MATERIALS AND METHODS

### 2.1 Animals

The experiments were performed on male ICR mice (80 ± 10 days old) (Shizuoka Laboratory Animal Center, Hamamatsu, Japan) housed in a forced‐air facility, which was maintained at 23°C and 50% relative humidity, and kept on a 12‐h light/dark cycle.^25^, ^38^ The mice were kept individually in single transparent cages measuring 23.5 × 16.5 × 12 cm, and were allowed water and rodent chow ad libitum. The experiments described in this report were approved by the Kyoto University Animal Experimentation Committee, and were conducted in accordance with the “Regulations on Animal Experimentation at Kyoto University” of the institution,^39^ which is based on the National Institutes of Health Guide for the Care and Use of Laboratory Animals. All efforts were made by trained personnel to minimize the pain experienced by the mice. No mice died during the experiments. All of the observations and evaluations were performed by a trained observer who was blinded to and not informed of the treatment conditions in advance. Each experimental group comprised 10 mice, based on previously published research and preliminary data.^25^, ^38^

### 2.2 Drug and stressor treatments

In the NC treatment groups, repeated subcutaneous (sc) doses of NC that caused both anxiety‐ and working memory impairment‐like behaviours effectively in mice were selected: a single sc dose of 0.8 mg/kg was administered daily for 4 days.^25^, ^38^ NC (Nacalai Tesque, Inc., Kyoto, Japan) was supplied in free‐base form at 95% purity, and was freshly dissolved in saline to a volume of 5 mL/kg immediately before each administration.^25^, ^38^, ^40^ For the stressor treatment groups, repeated immobilization (IM) stress treatments in which anxiogenic‐ and working memory impairment‐like effects similar to those of the NC treatments were selected: 10 min of IM, which was induced by placing the mouse in a narrow space (diameter about 12 cm) in a vinyl bag with some breathing holes, was performed once a day for 4 days.^25^, ^38^ Furthermore, to investigate the interactions between NC and IM, the behavioural alterations were examined in the NC plus IM group (NC‐IM group) which received the aforementioned sc dose of NC 10 min before the IM treatment once a day for 4 days.^25^, ^38^, ^41^

With respect to the HDAC inhibitors sodium butyrate (SB) and valproic acid (VA), the cannabinoid type 1 (CB1) agonist ACPA (arachidonylcyclopropylamide [AC]), the CB1 antagonist SR 141716A (*N*‐(piperidin‐1‐yl)‐5‐(4‐chlorophenyl)‐1‐(2,4‐dichlorophenyl)‐4‐methyl‐1H‐pyrazole‐3‐carboxamide hydrochloride [SR]), the TRPV1 agonist olvanil (OL), and the TRPV1 antagonist capsazepine (CZ), which were all purchased from Tocris Cookson Inc. (Ellisville, Missouri, USA), the data were collected and shown for the following intraperitoneal (ip) doses: 50, 100, and 200 mg/kg for SB; 200, 300, and 400 mg/kg for VA; 0.05, 0.2, and 1 mg/kg for AC; 0.5, 1, and 2 mg/kg for SR; 0.1, 1, and 2.5 mg/kg for OL; and 0.1, 1, and 5 mg/kg for CZ, based on previous data and preliminary experiments.^25^, ^42^, ^43^, ^44^, ^45^, ^46^, ^47^, ^48^, ^49^, ^50^ As ligands related to the ECB system, ligands for the CB1 receptors, the representative CB receptors expressed in the central nervous system, were selected.^25^, ^38^, ^42^, ^48^, ^49^, ^50^ The doses were selected from those that induced no toxic behavioural alterations (e.g., continuous suppression of locomotor activity) by themselves at the prescribed time point even when combined administration was repeated. The drugs were dissolved and diluted using a mixed solution of dimethylsulphoxide (DMSO) plus distilled water, and were administered in a total volume of 2.5 mL/kg 60 min (drugs except for VA) or 30 min (VA) before each NC, IM, or NC‐IM treatment. Furthermore, in the experiments examining the interacting role of HDAC inhibition (histone acetylation) with the ECB and/or TRPV1 system, each CB1 and/or TRPV1 ligand was used in combination with the effective HDAC inhibitors. In the HDAC inhibitor‐ or CB1/TRPV1 ligand‐only groups, an equivolume saline vehicle was injected instead of the NC, IM, or NC‐IM treatment. In the control group without any drug or stressor treatment (control group), a mixed vehicle solution of DMSO and distilled water was injected instead of the HDAC inhibitors or CB1/TRPV1 ligands, and then, an equivolume saline vehicle was injected instead of the NC, IM, or NC‐IM treatment. The drug and stressor treatments and each experimental session were performed 4–8 h after the beginning (8:00 a.m.) of the light cycle.

### 2.3 Elevated plus‐maze (EPM) test

Alterations in anxiety‐related behaviours were examined in the EPM test, using a cardboard apparatus that consisted of two opposite open arms 50 × 10 cm (length and width) and two enclosed arms 50 × 10 × 30 cm (length, width, and height), positioned 50 cm from the floor.^25^, ^38^, ^51^, ^52^, ^53^, ^54^ After the number of entries into open arms, the time spent on open arms (seconds) and the total number of entries into arms were evaluated (5‐min [300 s] test periods), the percentage of entries into open arms and the percentage of time spent on open arms were calculated as parameters of anxiety‐related behaviours. These evaluations were performed at the 2‐h time point after the last NC, IM, or NC‐IM treatment. At the beginning of each experimental session, each mouse was placed diagonally in the center platform of the maze, facing both the open and enclosed arms.

### 2.4 Y‐maze test

Alterations in working memory‐related behaviours were examined in the Y‐maze test using a cardboard apparatus that consisted of three enclosed arms 30 × 5 × 15 cm (length, width, and height) that converged on an equilateral triangular center platform (5 × 5 × 5 cm).^38^, ^55^, ^56^, ^57^ After the number of spontaneous alteration performance (SAP), which was defined as the number of successive triplet entry performances into each of the three arms without any repeated entries, and the total number of entries into arms were evaluated (8‐min test periods), the rate of spontaneous alteration performance (SAP rate) (%) was calculated as a parameter for the working memory‐related behaviours. These evaluations were performed at the 2‐h time point after the last NC, IM, or NC‐IM treatment. At the beginning of each experimental session, each mouse was placed in the center platform of the maze, facing all three arms immediately before the session.

### 2.5 Statistical analysis

The data were subjected to two‐ or three‐way analysis of variance (ANOVA) for each experiment.^25^, ^38^, ^58^ With respect to the experiments examining the NC‐ and/or IM‐induced anxiety‐ and working memory‐related behavioural alterations and the effects of each HDAC inhibitor, CB1 or TRPV1 ligand, a 2 × 2 or 4 × 4 factorial design was used. With respect to the experiments examining the interacting role of HDAC inhibition (histone acetylation) with the ECB and/or TRPV1 system, a 4 × 2 × 4 or 4 × 2 × 2 factorial design was used. For pairwise comparisons, Bonferroni post hoc tests were performed. All of the comparisons were performed using the statistical software “Excel Statistics” (Social Survey Research Information Co. Ltd., Tokyo, Japan).^25^, ^38^ *P* values less than 0.05 were considered to be statistically significant.

## 3 RESULTS

### 3.1 Mitigating effects of the HDAC inhibitor, CB1 agonist, or TRPV1 antagonist against NC and/or IM‐induced anxiety‐like behavioural alterations in the EPM test

**FIGURE 1:**
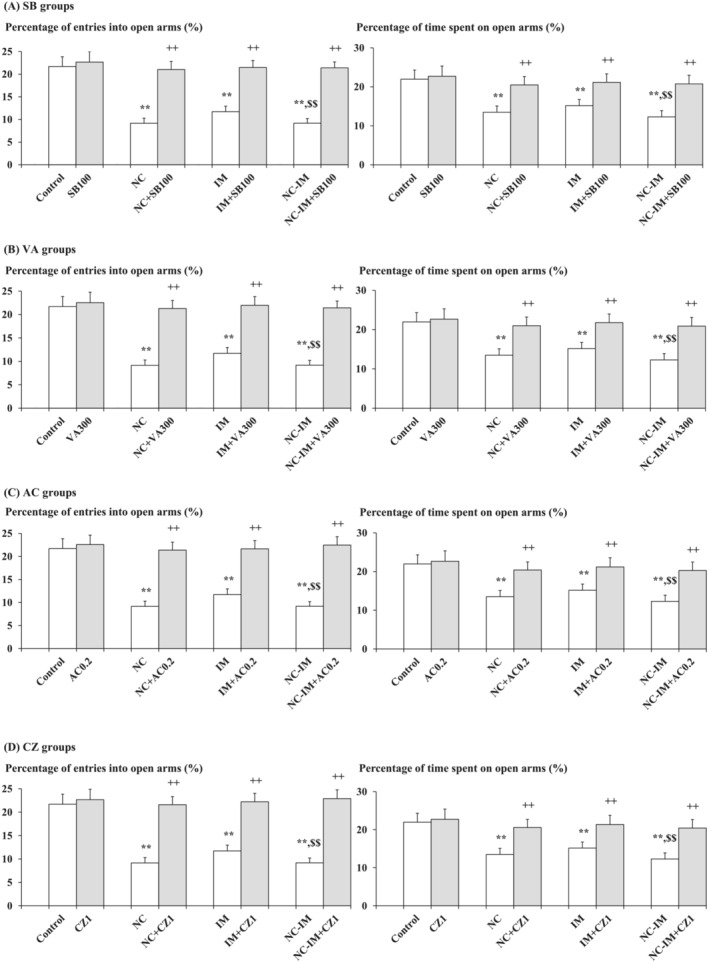
Mitigating effects of the HDAC inhibitors, CB1 agonist, or TRPV1 antagonist against anxiety‐like behaviours. The parameter values of the EPM test (percentages of entries into open arms and time spent on open arms) at the 2‐h time point after the last NC (0.8 mg/kg, sc) and/or IM (10 min) treatment are shown as means with standard deviation (SD) bars (*n* = 10) for each HDAC inhibitor (SB or VA), CB1 agonist (AC), or TRPV1 antagonist (CZ) cotreatment group (with each ip dose [mg/kg]), and statistical significance in posthoc tests is denoted using the symbols as defined below. The detailed data and statistical results have been included in Table S1. (A) SB (100 mg/kg, ip) cotreatment groups (SB groups); (B) VA (300 mg/kg, ip) cotreatment groups (VA groups); (C) AC (0.2 mg/kg, ip) cotreatment groups (AC groups); (D) CZ (1 mg/kg, ip) cotreatment groups (CZ groups). **P < 0.01: significant attenuation as compared with the control group; ++*P* < 0.01: significant increase as compared with the NC, IM, or NC‐IM group without any cotreatments; $$*P* < 0.01: significant attenuation as compared with the IM group without any cotreatments.

Consistent with previous studies,^25^, ^38^ anxiety‐like behavioural alterations in the EPM test, that is, significantly attenuated percentage of entries into open arms and significantly attenuated percentage of time spent on open arms as compared with the control group, were induced by repeated NC, IM, or NC‐IM treatments (Figure 1 and Table S1; NC, IM, or NC‐IM group). For the NC‐IM group, each parameter value was significantly attenuated as compared with the IM group (Figure 1 and Table S1; NC‐IM group). Using two‐way ANOVA, statistically significant main effects of NC (*F*(1, 36) = 268.59, *P* = 2.84 × 10^−18^ [*P* < 0.001] for the percentage of entries into open arms and *F*(1, 36) = 96.98, *P* = 9.34 × 10^−12^ [*P* < 0.001] for the percentage of time spent on open arms) and IM (*F*(1, 36) = 116.92, *P* = 7.39 × 10^−13^ [*P* < 0.001] for the percentage of entries into open arms and *F*(1, 36) = 48.32, *P* = 3.80 × 10^−8^ [*P* < 0.001] for the percentage of time spent on open arms) and statistically significant interactions between the NC and IM treatment in the NC‐IM group (*F*(1, 36) = 118.07, *P* = 6.45 × 10^−13^ [*P* < 0.001] for the percentage of entries into open arms and *F*(1, 36) = 23.68, *P* = 2.26 × 10^−5^ [*P* < 0.001] for the percentage of time spent on open arms) were observed (Table S7).

Against these anxiety‐like behavioural alterations, significant mitigating effects, that is, recoveries from both attenuated percentage of entries into open arms and attenuated percentage of time spent on open arms, were observed in the NC, IM, and NC‐IM groups cotreated with the HDAC inhibitor SB (50–200 mg/kg) or VA (200–400 mg/kg), the CB1 agonist AC (0.2–1 mg/kg), or the TRPV1 antagonist CZ (1–5 mg/kg) (Figure 1A–D and Table S1A‐D). This is consistent with the results of two‐way ANOVA revealing statistically significant interactions between the following combined treatments: NC and/or IM × SB (*F*(9, 144) = 21.73, *P* = 6.85 × 10^−23^ [*P* < 0.001] for the percentage of entries into open arms and *F*(9, 144) = 5.42, *P* = 2.16 × 10^−6^ [*P* < 0.001] for the percentage of time spent on open arms), NC and/or IM × VA (*F*(9, 144) = 22.12, *P* = 3.31 × 10^−23^ [*P* < 0.001] for the percentage of entries into open arms and *F*(9, 144) = 5.73, *P* = 8.85 × 10^−7^ [*P* < 0.001] for the percentage of time spent on open arms), NC and/or IM × AC (*F*(9, 144) = 30.53, *P* = 2.97 × 10^−29^ [*P* < 0.001] for the percentage of entries into open arms and *F*(9, 144) = 6.58, *P* = 7.70 × 10^−8^ [*P* < 0.001] for the percentage of time spent on open arms), and NC and/or IM × CZ (*F*(9, 144) = 29.13, *P* = 2.53 × 10^−28^ [*P* < 0.001] for the percentage of entries into open arms and F(9, 144) = 6.68, *P* = 5.78 × 10^−8^ [*P* < 0.001] for the percentage of time spent on open arms) (Table S7). In the groups cotreated with the CB1 antagonist SR or TRPV1 agonist OL, as well as in each HDAC inhibitor (SB or VA)‐ or CB1/TRPV1 ligand (AC, SR, OL or CZ)‐only group, no significant alterations as compared with the control group were observed for any parameter value under the present experimental conditions.

### 3.2 Mitigating effects of the HDAC inhibitor, TRPV1 agonist, or CB1 antagonist against NC and/or IM‐induced working memory impairment‐like behavioural alterations in the Y‐maze test

**FIGURE 2:**
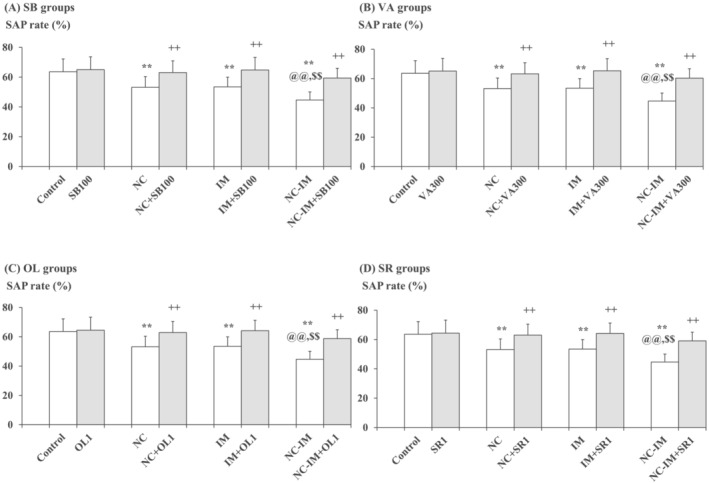
Mitigating effects of the HDAC inhibitors, TRPV1 agonist, or CB1 antagonist against working memory impairment‐like behaviours. The parameter values of the Y‐maze test (SAP rates) at the 2‐h time point after the last NC (0.8 mg/kg, sc) and/or IM (10 min) treatment are shown as means with SD bars (*n* = 10) for each HDAC inhibitor (SB or VA), TRPV1 agonist (OL), or CB1 antagonist (SR) cotreatment group (with each ip dose [mg/kg]), and statistical significance in post hoc tests is denoted using the symbols as defined below. The detailed data and statistical results have been included in Table S2. (A) SB (100 mg/kg, ip) cotreatment groups (SB groups); (B) VA (300 mg/kg, ip) cotreatment groups (VA groups); (C) OL (1 mg/kg, ip) cotreatment groups (OL groups); (D) SR (1 mg/kg, ip) cotreatment groups (SR groups). **P < 0.01: significant attenuation as compared with the control group; ++*P* < 0.01: significant increase as compared with the NC, IM, or NC‐IM group without any cotreatments; @@*P* < 0.01: significant attenuation as compared with the NC group without any cotreatments; $$*P* < 0.01: significant attenuation as compared with the IM group without any cotreatments.

Consistent with preliminary data,^38^ working memory impairment‐like behavioural plasticity in the Y‐maze test, that is, significantly attenuated rate of spontaneous alteration performance (SAP rate) (%) as compared with the control group, was induced by repeated NC, IM, or NC‐IM treatments (Figure 2 and Table S2; NC, IM, or NC‐IM group). For the NC‐IM group, the SAP rate was significantly attenuated as compared with the NC or IM group (Figure 2 and Table S2; NC‐IM group). Using two‐way ANOVA, statistically significant main effects of NC (*F*(1, 36) = 18.72, *P* = 1.15 × 10^−4^ [*P* < 0.001]) and IM (*F*(1, 36) = 17.62, *P* = 1.69 × 10^−4^ [*P* < 0.001]) were observed for the SAP rates (Table S8).

Against these working memory impairment‐like behavioural alterations, significant mitigating effects, that is, recoveries from the attenuated SAP rates, were observed in the NC, IM, and NC‐IM groups cotreated with the HDAC inhibitor SB (100–200 mg/kg) or VA (200–400 mg/kg), the TRPV1 agonist OL (1–2.5 mg/kg), or the CB1 antagonist SR (1–2 mg/kg) (Figure 2A–D and Table S2A‐D). This is consistent with the results of two‐way ANOVA revealing statistically significant main effects of SB (*F*(3, 144) = 12.45, *P* = 2.75 × 10^−7^ [*P* < 0.001]), VA (F(3, 144) = 13.32, *P* = 1.01 × 10^−7^ [*P* < 0.001]), OL (*F*(3, 144) = 12.36, *P* = 3.07 × 10^−7^ [*P* < 0.001]), and SR (*F*(3, 144) = 13.29, *P* = 1.05 × 10^−7^ [*P* < 0.001]) (Table S8). In the groups cotreated with the CB1 agonist AC or TRPV1 antagonist CZ, as well as in each HDAC inhibitor (SB or VA)‐ or CB1/TRPV1 ligand (AC, SR, OL or CZ)‐only group, no significant alterations in SAP rates as compared with the control group were observed under the present experimental conditions.

### 3.3 Anxiety‐related interacting effects between the mitigating drug (HDAC inhibitor, CB1 agonist, or TRPV1 antagonist) and CB1 antagonist with or without additional TRPV1 antagonist

**FIGURE 3:**
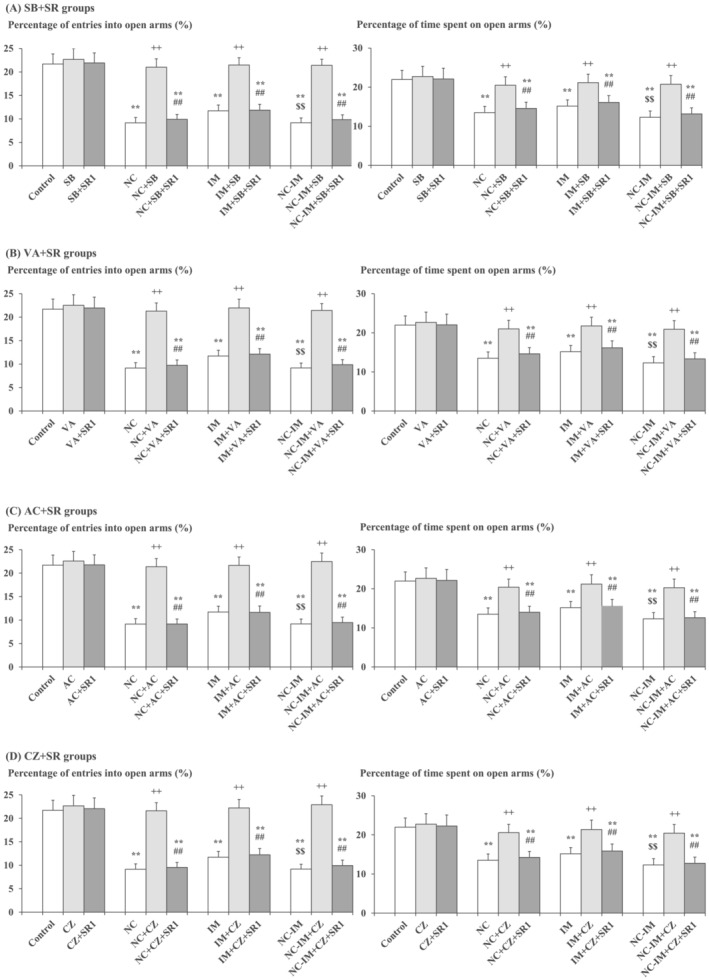
Interacting effects between the CB1 antagonist and mitigating (anxiolytic‐like) drug (i.e., HDAC inhibitor, CB1 agonist, or TRPV1 antagonist) against anxiety‐like behavioural alterations caused by NC and/or IM. The parameter values of the EPM test at the 2‐h time point after the last NC (0.8 mg/kg, sc) or IM (10 min) treatment are shown as means with SD bars (*n* = 10) for each mitigating drug plus CB1 antagonist group (with each ip dose [mg/kg]), and statistical significance in post hoc tests is denoted using the symbols as defined below. The detailed data and statistical results have been included in Table S3. (A) SB (100 mg/kg, ip) plus SR (1 mg/kg, ip) groups (SB + SR groups); (B) VA (300 mg/kg, ip) plus SR (1 mg/kg, ip) groups (VA + SR groups); (C) AC (0.2 mg/kg, ip) plus SR (1 mg/kg, ip) groups (AC + SR groups); (D) CZ (1 mg/kg, ip) plus SR (1 mg/kg, ip) groups (CZ + SR groups). **P < 0.01: significant attenuation as compared with the control group; ++*P* < 0.01: significant increase as compared with the NC, IM, or NC‐IM group without any cotreatments; $$*P* < 0.01: significant attenuation as compared with the IM group without any cotreatments; ##*P* < 0.01: significant attenuation as compared with the NC, IM, or NC‐IM group cotreated with the efficacious HDAC inhibitor, CB1 agonist, or TRPV1 antagonist.

Based on the data shown in Figure 1 and Table S1, interactions with the CB1 antagonist SR (0.5–2 mg/kg) were examined for the most effective dose of the HDAC inhibitor SB (100 mg/kg) or VA (300 mg/kg), as well as for the most effective dose of the CB1 agonist AC (0.2 mg/kg), to investigate the interacting roles of HDAC inhibitors with the ECB system. Against the “anxiolytic‐like” effects of SB or VA, as well as against those effects of AC, significant attenuating effects of SR (1–2 mg/kg) were observed for each parameter in the NC, IM, and NC‐IM groups (Figure 3A–C and Table S3A‐C). Moreover, even against the most anxiolytic‐like dose of the TRPV1 antagonist CZ (1 mg/kg), significant attenuating effects were observed for SR (1–2 mg/kg) in the NC, IM, and NC‐IM groups (Figure 3D and Table S3D). The data in Figure 3 and Table S3 are consistent with the results of three‐way ANOVA revealing statistically significant interactions for the following combined treatments: NC and/or IM × SB × SR (*F*(9, 288) = 15.96, *P* = 3.60 × 10^−21^ [*P* < 0.001] for the percentage of entries into open arms and *F*(9, 288) = 3.06, *P* = 0.00162 [*P* < 0.01] for the percentage of time spent on open arms), NC and/or IM × VA × SR (*F*(9, 288) = 15.63, *P* = 9.24 × 10^−21^ [*P* < 0.001] for the percentage of entries into open arms and *F*(9, 288) = 3.21, *P* = 0.00101 [*P* < 0.01] for the percentage of time spent on open arms), NC and/or IM × AC × SR (*F*(9, 288) = 16.89, *P* = 2.55 × 10^−22^ [*P* < 0.001] for the percentage of entries into open arms and *F*(9, 288) = 2.85, *P* = 0.00308 [*P* < 0.01] for the percentage of time spent on open arms), and NC and/or IM × CZ × SR (*F*(9, 288) = 17.16, *P* = 1.20 × 10^−22^ [*P* < 0.001] for the percentage of entries into open arms and *F*(9, 288) = 2.76, *P* = 0.00410 [*P* < 0.01] for the percentage of time spent on open arms) (Table S7). In each HDAC inhibitor (or CB1 agonist AC or TRPV1 antagonist CZ) plus CB1 antagonist SR‐only group, no significant alterations as compared with the control group were observed for any parameter value under the present experimental conditions.

**FIGURE 4:**
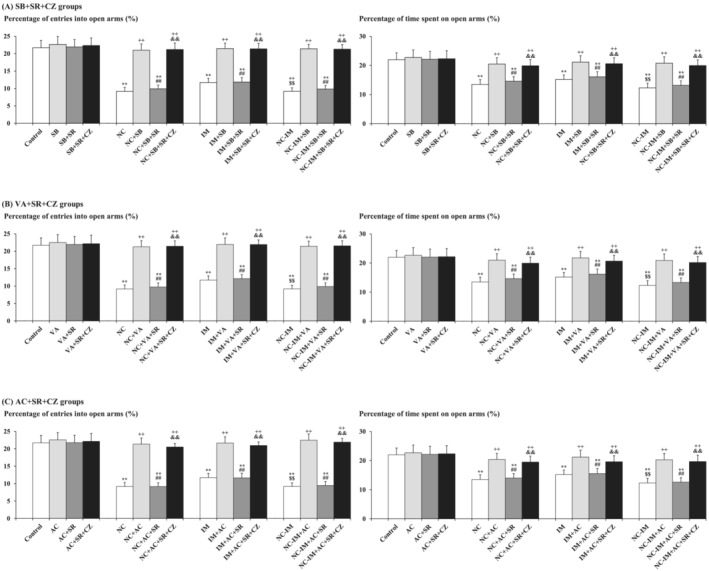
Counteraction caused by the TRPV1 antagonist against attenuating effects of the CB1 antagonist on HDAC inhibitor‐ or CB1 agonist‐induced anxiolytic‐like behavioural alterations in the NC and/or IM treatment groups. The parameter values of the EPM test at the 2‐h time point after the last NC (0.8 mg/kg, sc) and/or IM (10 min) treatment are shown as means with SD bars (*n* = 10) for each anxiolytic‐like drug (HDAC inhibitor or CB1 agonist) plus CB1 antagonist cotreatment group with or without additional TRPV1 antagonist (with each ip dose [mg/kg]), and statistical significance in post hoc tests is denoted using the symbols as defined below. The detailed data and statistical results have been included in Table S4. (A) SB (100 mg/kg, ip) plus SR (1 mg/kg, ip) cotreatment groups with (or without) additional CZ (1 mg/kg, ip) (SB + SR + CZ groups); (B) VA (300 mg/kg, ip) plus SR (1 mg/kg, ip) cotreatment groups with (or without) additional CZ (1 mg/kg, ip) (VA + SR + CZ groups); (C) AC (0.2 mg/kg, ip) plus SR (1 mg/kg, ip) cotreatment groups with (or without) additional CZ (1 mg/kg, ip) (AC + SR + CZ groups). **P < 0.01: significant attenuation as compared with the control group; ++*P* < 0.01: significant increase as compared with the NC, IM, or NC‐IM group without any cotreatments; $$*P* < 0.01: significant attenuation as compared with the IM group without any cotreatments; ##*P* < 0.01: significant attenuation as compared with the NC, IM, or NC‐IM group cotreated with the efficacious (anxiolytic‐like) HDAC inhibitor or CB1 agonist; &&*P* < 0.01: significant increase as compared with the NC, IM, or NC‐IM group cotreated with the mitigating drug (HDAC inhibitor or CB1 agonist) plus SR.

In the next experiment, additional effects of the TRPV1 antagonist CZ were examined in the NC and/or IM groups cotreated with the most effective doses of the HDAC inhibitor (SB or VA) plus SR (CB1 antagonist), or AC (CB1 agonist) plus SR, to further elucidate the combined roles of the ECB plus TRPV1 systems. By combining CZ (1 mg/kg) with SB (100 mg/kg) plus SR (1 mg/kg), VA (300 mg/kg) plus SR (1 mg/kg), or AC (0.2 mg/kg) plus SR (1 mg/kg), the “antianxiolytic‐like” effects of SR (Figure 3 and Table S3) were significantly counteracted as compared with non‐CZ groups (Figure 4A–C and Table S4A‐C). This is consistent with the results of three‐way ANOVA revealing statistically significant interactions between the following combined treatments: SB plus SR × CZ (*F*(1, 144) = 5.54, *P* = 0.0199 [*P* < 0.05] for the percentage of entries into open arms and *F*(1, 144) = 3.98, *P* = 0.0480 [*P* < 0.05] for the percentage of time spent on open arms), VA plus SR × CZ (*F*(1, 144) = 3.96, *P* = 0.0484 [*P* < 0.05] for the percentage of entries into open arms and *F*(1, 144) = 4.07, *P* = 0.0454 [*P* < 0.05] for the percentage of time spent on open arms), and AC plus SR × CZ (*F*(1, 144) = 4.18, *P* = 0.0427 [*P* < 0.05] for the percentage of entries into open arms and *F*(1, 144) = 3.92, *P* = 0.0496 [*P* < 0.05] for the percentage of time spent on open arms) (Table S7). In each HDAC inhibitor (or CB1 agonist AC) plus CB1 antagonist SR plus TRPV1 antagonist CZ‐only group, no significant alterations as compared with the control group were observed for any parameter value under the present experimental conditions.

### 3.4 Working memory impairment‐related interacting effects between the mitigating drug (HDAC inhibitor, TRPV1 agonist, or CB1 antagonist) and TRPV1 antagonist with or without additional CB1 antagonist

**FIGURE 5:**
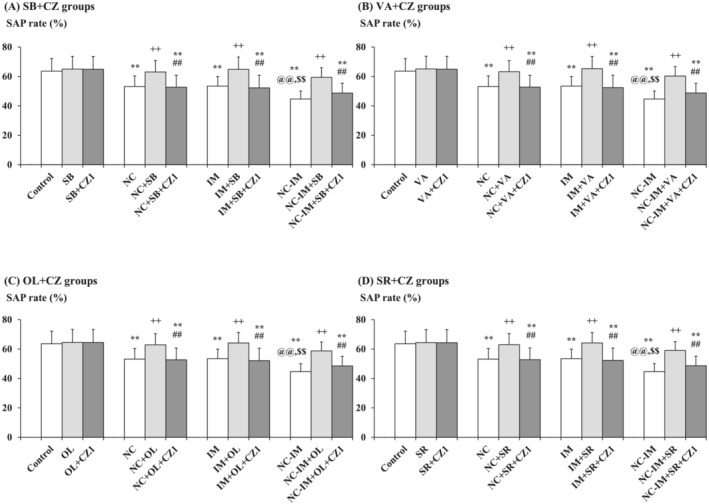
Interacting effects between the TRPV1 antagonist and mitigating (working memory improving‐like) drug (i.e., HDAC inhibitor, TRPV1 agonist, or CB1 antagonist) against working memory impairment‐like behavioural alterations caused by NC and/or IM. The parameter values of the Y‐maze test at the 2‐h time point after the last NC (0.8 mg/kg, sc) or IM (10 min) treatment are shown as means with SD bars (*n* = 10) for each mitigating drug plus TRPV1 antagonist group (with each ip dose [mg/kg]), and statistical significance in post hoc tests is denoted using the symbols as defined below. The detailed data and statistical results have been included in Table S5. (A) SB (100 mg/kg, ip) plus CZ (1 mg/kg, ip) groups (SB + CZ groups); (B) VA (300 mg/kg, ip) plus CZ (1 mg/kg, ip) groups (VA + CZ groups); (C) OL (1 mg/kg, ip) plus CZ (1 mg/kg, ip) groups (OL + CZ groups); (D) SR (1 mg/kg, ip) plus CZ (1 mg/kg, ip) groups (SR + CZ groups). **P < 0.01: significant attenuation as compared with the control group; ++*P* < 0.01: significant increase as compared with the NC, IM, or NC‐IM group without any cotreatments; @@*P* < 0.01: significant attenuation as compared with the NC group without any cotreatments; $$*P* < 0.01: significant attenuation as compared with the IM group without any cotreatments; ##*P* < 0.01: significant attenuation as compared with the NC, IM, or NC‐IM group cotreated with the efficacious HDAC inhibitor, TRPV1 agonist, or CB1 antagonist.

Based on the data shown in Figure 2 and Table S2, interactions with the TRPV1 antagonist CZ (0.1–5 mg/kg) were examined for the most effective dose of the HDAC inhibitor SB (100 mg/kg) or VA (200 mg/kg), as well as for the most effective dose of the TRPV1 agonist OL (1 mg/kg), to investigate the interacting roles of HDAC inhibitors with the TRPV1 system. Against the “working memory improving‐like” effects of SB or VA, as well as against those effects of OL, significant attenuating effects were observed for CZ (1–5 mg/kg) in the NC, IM, and NC‐IM groups (Figure 5A–C and Table S5A‐C). Moreover, even against the most efficacious dose of the CB1 antagonist SR (1 mg/kg), significant attenuating effects were observed for CZ (1–5 mg/kg) in the NC, IM, and NC‐IM groups (Figure 5D and Table S5D). The data of SAP rates in Figure 5 and Table S5 are consistent with the results of three‐way ANOVA revealing statistically significant interactions for the following combined treatments: SB × CZ (*F*(3, 288) = 4.74, *P* = 0.00304 [*P* < 0.01]), VA × CZ (*F*(3, 288) = 4.92, *P* = 0.00240 [*P* < 0.01]), OL × CZ (*F*(3, 288) = 4.46, *P* = 0.00444 [*P* < 0.01]), and SR × CZ (*F*(3, 288) = 4.51, *P* = 0.00413 [*P* < 0.01]) (Table S8). In each HDAC inhibitor (or TRPV1 agonist OL or CB1 antagonist SR) plus TRPV1 antagonist CZ‐only group, no significant alterations in SAP rates as compared with the control group were observed under the present experimental conditions.

**FIGURE 6:**
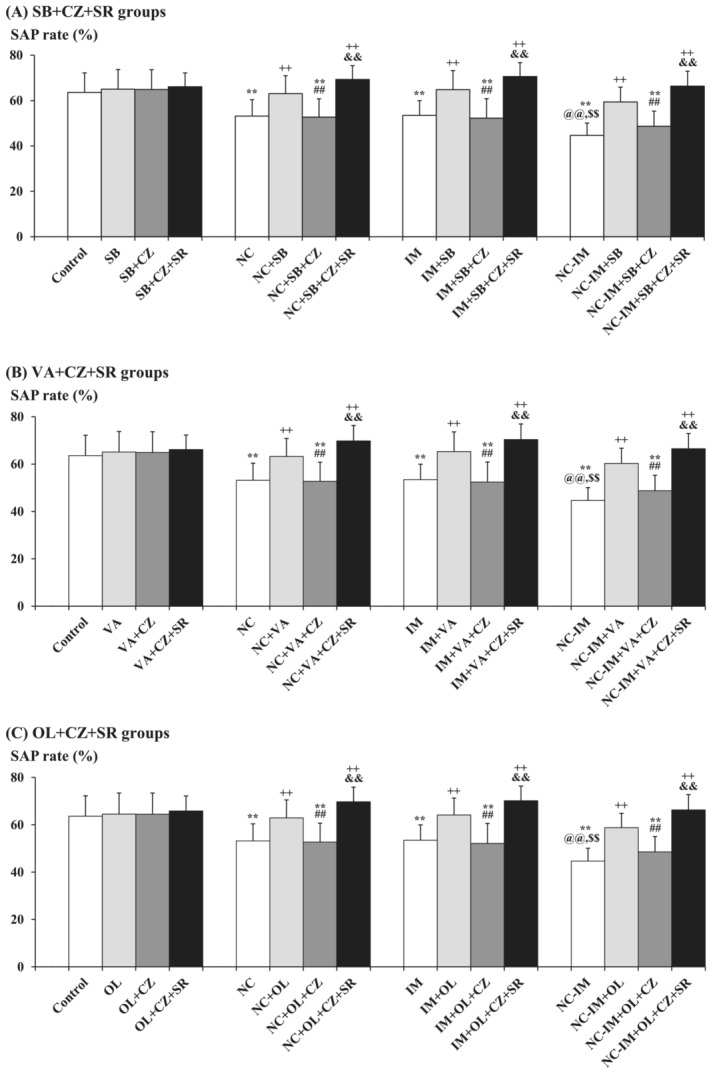
Counteraction caused by the CB1 antagonist against attenuating effects of the TRPV1 antagonist on HDAC inhibitor‐ or TRPV1 agonist‐induced working memory improving‐like behavioural alterations in the NC and/or IM treatment groups. The parameter values of the Y‐maze test at the 2‐h time point after the last NC (0.8 mg/kg, sc) and/or IM (10 min) treatment are shown as means with SD bars (*n* = 10) for each working memory improving‐like drug (HDAC inhibitor or TRPV1 agonist) plus TRPV1 antagonist cotreatment group with or without additional CB1 antagonist (with each ip dose [mg/kg]), and statistical significance in post hoc tests is denoted using the symbols as defined below. The detailed data and statistical results have been included in Table S6. (A) SB (100 mg/kg, ip) plus CZ (1 mg/kg, ip) cotreatment groups with (or without) additional SR (1 mg/kg, ip) (SB + CZ + SR groups); (B) VA (300 mg/kg, ip) plus CZ (1 mg/kg, ip) cotreatment groups with (or without) additional SR (1 mg/kg, ip) (VA + CZ + SR groups); (C) OL (1 mg/kg, ip) plus CZ (1 mg/kg, ip) cotreatment groups with (or without) additional SR (1 mg/kg, ip) (OL + CZ + SR groups). **P < 0.01: significant attenuation as compared with the control group; ++*P* < 0.01: significant increase as compared with the NC, IM, or NC‐IM group without any cotreatments; @@*P* < 0.01: significant attenuation as compared with the NC group without any cotreatments; $$*P* < 0.01: significant attenuation as compared with the IM group without any cotreatments; ##*P* < 0.01: significant attenuation as compared with the NC, IM, or NC‐IM group cotreated with the efficacious (working memory improving‐like) HDAC inhibitor or TRPV1 agonist; &&*P* < 0.01: significant increase as compared with the NC, IM, or NC‐IM group cotreated with the mitigating drug (HDAC inhibitor or TRPV1 agonist) plus CZ.

In the next experiment, additional effects of the CB1 antagonist SR were examined in the NC and/or IM groups cotreated with the most effective doses of the HDAC inhibitor (SB or VA) plus CZ (TRPV1 antagonist), or OL (TRPV1 agonist) plus CZ, to further elucidate the combined roles of the TRPV1 plus ECB systems. By combining SR (1 mg/kg) with SB (100 mg/kg) plus CZ (1 mg/kg), VA (300 mg/kg) plus CZ (1 mg/kg), or OL (1 mg/kg) plus CZ (1 mg/kg), the “antiworking memory improving‐like” effects of CZ on SAP rates (Figure 5 and Table S5) were significantly counteracted as compared with non‐SR groups (Figure 6A–C and Table S6A‐C). This is consistent with the results of three‐way ANOVA revealing statistically significant interactions between the following combined treatments: SB plus CZ × SR (*F*(1, 144) = 3.95, *P* = 0.0488 [*P* < 0.05]), VA plus CZ × SR (*F*(1, 144) = 4.00, *P* = 0.0474 [*P* < 0.05]), and OL plus CZ × SR (*F*(1, 144) = 4.08, *P* = 0.0452 [*P* < 0.05]) (Table S8). In each HDAC inhibitor (or TRPV1 agonist OL) plus TRPV1 antagonist CZ plus CB1 antagonist SR‐only group, no significant alterations in SAP rates as compared with the control group were observed under the present experimental conditions.

## 4 DISCUSSION

### 4.1 NC‐ and/or IM‐induced anxiety‐ and working memory impairment‐like behavioural alterations and mitigating effects of HDAC inhibitors

In the NC group receiving repeated treatments of NC, as well as in the IM group, anxiety‐like behavioural alterations in the EPM test and working memory impairment‐like behavioural alterations in the Y‐maze test were observed, which supports the findings from previous studies.^25^, ^38^ Although the opposite effects on anxiety and working memory have been reported for NC depending on the rodent experimental condition,^11^, ^12^, ^13^, ^14^ anxiogenic‐ and working memory impairment‐like effects like those observed for the IM treatment were induced for the NC treatment in the present paradigm, for which a pivotal involvement of dysfunctional neural circuits associated with the nicotinic cholinergic system has been suggested.^59^, ^60^, ^61^ In cooperation with such neural circuit mechanisms, the contribution of altered activity of stress‐related neurotransmitter systems (e.g., dopaminergic system) has been reported for the anxiety‐ and working memory‐related behavioural effects of NC.^60^, ^62^, ^63^, ^64^ Regarding interactions between NC and IM in the NC‐IM group, significant enhancements of both anxiogenic‐ and working memory impairment‐like effects were induced by NC. In spite of the mitigating effects of NC against stressor‐induced emotional and cognitive dysfunction in certain treatment conditions accompanied by a protective influence on the relevant stress‐related neurotransmitter systems, biochemical responses and synaptic plasticity,^65^, ^66^, ^67^, ^68^ synergistic behavioural impairments like those reported in some studies^25^, ^38^, ^69^, ^70^ were observed. Here, excessive augmentation in the activity of the hypothalamic–pituitary–adrenal (HPA) axis and immediate early genes associated with neural circuit mechanisms^69^, ^71^, ^72^ may be implicated.

The NC‐ and/or IM‐induced anxiety‐ and working memory impairment‐like behavioural alterations were mitigated by the HDAC inhibitors SB and VA, consistent with recent studies demonstrating histone acetylation‐related therapeutic effects of these representative HDAC inhibitors.^73^, ^74^, ^75^, ^76^, ^77^ Several studies have reported the favourable influence of HDAC inhibitor‐induced histone acetylation in the control of stress‐related neural circuits.^78^, ^79^ Moreover, histone acetylation at the regions of genes encoding NC‐ and/or stress‐related neurotransmitter systems has contributed to the alleviation of relevant behavioural dysfunction.^80^, ^81^, ^82^ Although the enhancement of stressor‐like effects accompanied by impaired behavioural plasticity has been suggested at high doses of SB and VA,^83^, ^84^ controlled HDAC inhibition seems to mitigate the NC‐ and/or IM‐induced anxiety‐ and working memory impairment‐like behaviours.

### 4.2 Effects of ECB and/or TRPV1 system‐related ligands against NC‐ and/or IM‐induced anxiety‐ and working memory impairment‐like behavioural alterations and epigenetic interactions with HDAC inhibitors

In the present study, the CB1 agonist AC and the TRPV1 antagonist CZ mitigated the NC‐ and/or IM‐induced anxiety‐like behavioural alterations. Against the working memory impairment‐like behavioural alterations, mitigating effects were induced by the TRPV1 agonist OL and the CB1 antagonist SR. The effects of CB1 ligands are consistent with various rodent data,^51^, ^85^, ^86^, ^87^, ^88^, ^89^, ^90^ and support the aforementioned studies on the neuroanatomical and functional interactions between the ECB and NC‐ and/or stress‐related neurotransmitter systems.^26^, ^27^, ^28^, ^29^, ^30^ However, the anxiolytic‐like effects of AC were limited (efficacious at 0.05–0.2 mg/kg), and differential involvement of the complementary neurotransmitter systems represented by the TRPV1 system^91^, ^92^, ^93^ could be assumed. Like AC, CZ mitigated the anxiogenic‐like effects of NC and/or IM, consistent with previous experimental studies using CZ‐like TRPV1 antagonists,^46^, ^94^, ^95^, ^96^ and altered facilitation of ECB system‐related complementary neurotransmission^97^, ^98^, ^99^ has been suggested. Intriguingly, the anxiolytic‐like effects of the TRPV1 antagonist CZ, like the effects of the CB1 agonist AC, were attenuated by the CB1 antagonist SR. Moreover, the antianxiolytic‐like effects of SR against AC were further counteracted by CZ. From these results, it can be hypothesized that the mutual control of the ECB and TRPV1 systems^91^, ^100^, ^101^ may crucially affect the manifestation of anxiety‐related behavioural plasticity.

Against working memory impairment‐like behaviours, unlike against anxiety‐like behaviours, ameliorating effects were induced by the CB1 antagonist SR, for which combined involvement of the complementary neurotransmitter systems,^102^, ^103^, ^104^ along with specific biochemical/neurophysiological modifications in memory‐related brain regions,^105^, ^106^ could be assumed. Such mitigating effects were also exerted by the TRPV1 agonist OL, consistent with previous studies using TRPV1 agonists,^107^, ^108^ and simultaneous modulation of ECB system‐related complementary neurotransmission observed in anxiety‐related behavioural plasticity^97^, ^98^, ^99^ seemed to be implicated. Moreover, the recovering effects of SR, like the effects of OL, were attenuated by the TRPV1 antagonist CZ, and the antirecovering effects of CZ against OL were further counteracted by SR, which may reflect the mutually controlled activation of the ECB and TRPV1 systems in memory‐related behavioural plasticity.^109^, ^110^, ^111^ Although the detailed mechanisms underlying the discrepancy in the efficacy of CB1/TRPV1 ligands between the anxiety‐ and working memory impairment‐related behaviours remain elusive, the differences in the relevant brain regions,^112^, ^113^, ^114^, ^115^ along with polymodal regulation of synaptic plasticity involved in the two neurotransmitter systems,^116^, ^117^ seemed to contribute, and this may be controlled subtly at the epigenetic level.

Like the effects induced by AC and CZ, the anxiolytic‐like effects of the HDAC inhibitors SB and VA against the NC and/or IM treatment were significantly attenuated by the CB1 antagonist SR, which was further counteracted by the TRPV1 antagonist CZ. On the other hand, the working memory improving‐like effects of the HDAC inhibitors SB and VA were significantly attenuated by CZ, which was further counteracted by SR. From such results, the involvement of HDAC inhibitor‐induced histone acetylation in the interacting influence of the ECB and TRPV1 system‐related ligands on the anxiogenic‐ and working memory impairment‐like behaviours was hypothesized. The antianxiolytic‐like effects of SR against SB and VA which were further counteracted by CZ, as well as the antimemory improving‐like effects of CZ which were further counteracted by SR, may reflect some mediatory roles of histone acetylation in the interplay between the ECB and TRPV1 systems. A number of studies have suggested the contribution of reduced histone acetylation to both stress (or drug)‐related impaired emotional/cognitive behaviours and disturbed function of either the ECB or TRPV1 system.^118^, ^119^, ^120^, ^121^, ^122^, ^123^, ^124^ Nevertheless, few studies have discussed the role of HDAC inhibitor‐induced histone acetylation in the interacting behavioural influence of the ECB and TRPV1 systems.^125^ Recently, the cooperative epigenetic involvement of the ECB and TRPV1 systems in the pathogenesis of various diseases, including aberrant inflammatory responses observed in COVID‐19, has been suggested.^126^, ^127^ Controlled histone acetylation may contribute to normalized crosstalk between the ECB and TRPV1 systems, which could regulate both emotional and cognitive behaviours, and develop potential therapeutic interventions.

### 4.3 Conclusion

In summary, the present results showed the protective effects of HDAC inhibitors (SB and VA) against the NC‐ and/or IM‐induced anxiety‐ and working memory impairment‐like behavioural alterations, for which the anxiolytic‐like effects were mimicked by the CB1 agonist AC or the TRPV1 antagonist CZ, whereas the working memory improving‐like effects were mimicked by the CB1 antagonist SR or the TRPV1 agonist OL. Moreover, a reversing influence was observed for SR against the anxiolytic‐like effects of the HDAC inhibitors, AC or CZ, which was further counteracted by CZ. On the other hand, a reversing influence was observed for CZ against the working memory improving‐like effects of the HDAC inhibitors, SR or OL, which was further counteracted by SR. These findings of the interrelated involvement may suggest a therapeutically important role of the HDAC inhibitor‐induced epigenetic histone acetylation in the interplay between the ECB and TRPV1 systems against aberrant behavioural plasticity.

## AUTHOR CONTRIBUTIONS

TH designed and performed research. TH also analysed data and wrote the paper.

## CONFLICT OF INTEREST STATEMENT

The author has no competing interests to declare.

## Supporting information


**Table S1.** Data for the mitigating effects of the HDAC inhibitors, CB1 agonist or TRPV1 antagonist against anxiety‐like behaviours, which are summarized and depicted in Figure 1.
**Table S2.** Data for the mitigating effects of the HDAC inhibitors, TRPV1 agonist or CB1 antagonist against working memory impairment‐like behaviours, which are summarized and depicted in Figure 2.
**Table S3.** Data for the interacting effects between the CB1 antagonist and mitigating (anxiolytic‐like) drug (i.e., HDAC inhibitor, CB1 agonist or TRPV1 antagonist) against anxiety‐like behavioural alterations caused by NC and/or IM, which are summarized and depicted in Figure 3.
**Table S4.** Data for the counteraction caused by the TRPV1 antagonist against attenuating effects of the CB1 antagonist on HDAC inhibitor‐ or CB1 agonist‐induced anxiolytic‐like behavioural alterations in the NC and/or IM treatment groups, which are depicted in Figure 4.
**Table S5.** Data for the interacting effects between the TRPV1 antagonist and mitigating (working memory improving‐like) drug (i.e., HDAC inhibitor, TRPV1 agonist or CB1 antagonist) against working memory impairment‐like behavioural alterations caused by NC and/or IM, which are summarized and depicted in Figure 5.
**Table S6.** Data for the counteraction caused by the CB1 antagonist against attenuating effects of the TRPV1 antagonist on HDAC inhibitor‐ or TRPV1 agonist‐induced working memory improving‐like behavioural alterations in the NC and/or IM treatment groups, which are depicted in Figure 6.
**Table S7.** Results of two‐ or three‐way analysis of variance (ANOVA) for the parameter values of anxiety‐related behavioural plasticity in the EPM test (percentages of entries into open arms and time spent on open arms).
**Table S8.** Results of two‐ or three‐way analysis of variance (ANOVA) for the parameter values of working memory impairment‐related behavioural plasticity in the Y maze test (SAP rate).

## Data Availability

The data that support the findings of this study are available from the corresponding authors upon reasonable request.
