# Supplementary material for: Interrelated involvement of the endocannabinoid/endovanilloid (TRPV1) systems and epigenetic processes in anxiety‐ and working memory impairment‐related behavioural effects of nicotine as a stressor
Source: Addict Biol. 2024 Jul 4;29(7):10.1111/adb.13421. doi: 10.1111/adb.13421 (PMC11222983; doi:10.1111/adb.13421)
Supplement: Supplementary file 1 — Table S1. Data for the mitigating effects of the HDAC inhibitors, CB1 agonist or TRPV1 antagonist against anxiety‐like behaviours, which are summarized and depicted in Figure 1. Table S2. Data for the mitigating effects of the HDAC inhibitors, TRPV1 agonist or CB1 antagonist against working memory impairment‐like behaviours, which are summarized and depicted in Figure 2. Table S3. Data for the interacting effects between the CB1 antagonist and mitigating (anxiolytic‐like) drug (i.e., HDAC inhibitor, CB1 agonist or TRPV1 antagonist) against anxiety‐like behavioural alterations caused by NC and/or IM, which are summarized and depicted in Figure 3. Table S4. Data for the counteraction caused by the TRPV1 antagonist against attenuating effects of the CB1 antagonist on HDAC inhibitor‐ or CB1 agonist‐induced anxiolytic‐like behavioural alterations in the NC and/or IM treatment groups, which are depicted in Figure 4. Table S5. Data for the interacting effects between the TRPV1 antagonist and mitigating (working memory improving‐like) drug (i.e., HDAC inhibitor, TRPV1 agonist or CB1 antagonist) against working memory impairment‐like behavioural alterations caused by NC and/or IM, which are summarized and depicted in Figure 5. Table S6. Data for the counteraction caused by the CB1 antagonist against attenuating effects of the TRPV1 antagonist on HDAC inhibitor‐ or TRPV1 agonist‐induced working memory improving‐like behavioural alterations in the NC and/or IM treatment groups, which are depicted in Figure 6. Table S7. Results of two‐ or three‐way analysis of variance (ANOVA) for the parameter values of anxiety‐related behavioural plasticity in the EPM test (percentages of entries into open arms and time spent on open arms). Table S8. Results of two‐ or three‐way analysis of variance (ANOVA) for the parameter values of working memory impairment‐related behavioural plasticity in the Y maze test (SAP rate). [file ADB-29--s001.docx]

**SUPPLEMENTARY TABLES**

**TABLE S1.** Data for the mitigating effects of the HDAC inhibitors, CB1 agonist or TRPV1 antagonist against anxiety-like behaviors, which are summarized and depicted in FIGURE 1.

| (A) SB groups | | |
| --- | --- | --- |
| Groups | Percentage of entries into open arms | Percentage of time spent on open arms |
| Control | 21.7±2.14 | 22.0±2.35 |
| SB50 | 22.4±2.16 | 22.2±2.52 |
| SB100 | 22.7±2.31 | 22.7±2.62 |
| SB200 | 22.7±2.50 | 22.8±2.63 |
| NC | 9.16±1.14 ** | 13.5±1.61 ** |
| NC+SB50 | 19.8±1.61 *,++ | 19.9±2.06 *,++ |
| NC+SB100 | 21.0±1.78 ++ | 20.5±2.16 ++ |
| NC+SB200 | 20.3±1.85 ++ | 20.2±2.16 ++ |
| IM | 11.7±1.23 ** | 15.2±1.59 ** |
| IM+SB50 | 20.8±1.36 ++ | 20.4±2.14 ++ |
| IM+SB100 | 21.5±1.54 ++ | 21.2±2.17 ++ |
| IM+SB200 | 21.1±1.56 ++ | 20.8±2.18 ++ |
| NC-IM | 9.18±1.03 **,$$ | 12.3±1.61 **,$$ |
| NC-IM+SB50 | 19.5±1.16 *,++ | 19.7±2.07 *,++ |
| NC-IM+SB100 | 21.4±1.30 ++ | 20.8±2.21 ++ |
| NC-IM+SB200 | 20.0±1.32 *,++ | 19.8±2.21 *,++ |
| (B) VA groups | | |
| Groups | Percentage of entries into open arms | Percentage of time spent on open arms |
| Control | 21.7±2.14 | 22.0±2.35 |
| VA200 | 22.2±2.15 | 22.2±2.52 |
| VA300 | 22.5±2.25 | 22.7±2.62 |
| VA400 | 22.6±2.38 | 22.7±2.64 |
| NC | 9.16±1.14 ** | 13.5±1.61 ** |
| NC+VA200 | 20.1±1.35 ++ | 19.9±1.96 *,++ |
| NC+VA300 | 21.3±1.73 ++ | 21.0±2.21 ++ |
| NC+VA400 | 20.4±1.74 ++ | 20.4±2.24 ++ |
| IM | 11.7±1.23 ** | 15.2±1.59 ** |
| IM+VA200 | 21.1±1.56 ++ | 20.5±2.12 ++ |
| IM+VA300 | 22.0±1.86 ++ | 21.8±2.22 ++ |
| IM+VA400 | 21.4±1.91 ++ | 21.1±2.24 ++ |
| NC-IM | 9.18±1.03 **,$$ | 12.3±1.61 **,$$ |
| NC-IM+VA200 | 20.0±1.35 ++ | 19.7±2.08 *,++ |
| NC-IM+VA300 | 21.4±1.45 ++ | 20.9±2.21 ++ |
| NC-IM+VA400 | 20.0±1.49 ++ | 19.8±2.21 *,++ |
| (C) AC groups | | |
| Groups | Percentage of entries into open arms | Percentage of time spent on open arms |
| Control | 21.7±2.14 | 22.0±2.35 |
| AC0.05 | 22.1±2.02 | 22.2±2.55 |
| AC0.2 | 22.6±2.06 | 22.7±2.68 |
| AC1 | 22.7±2.10 | 22.7±2.70 |
| NC | 9.16±1.14 ** | 13.5±1.61 ** |
| NC+AC0.05 | 9.97±1.55 ** | 14.7±1.66 ** |
| NC+AC0.2 | 21.4±1.73 ++ | 20.4±2.08 ++ |
| NC+AC1 | 20.3±1.77 ++ | 20.3±2.10 ++ |
| IM | 11.7±1.23 ** | 15.2±1.59 ** |
| IM+AC0.05 | 12.6±1.29 ** | 16.4±1.77 ** |
| IM+AC0.2 | 21.7±1.78 ++ | 21.2±2.37 ++ |
| IM+AC1 | 21.0±1.78 ++ | 20.8±2.39 ++ |
| NC-IM | 9.18±1.03 **,$$ | 12.3±1.61 **,$$ |
| NC-IM+AC0.05 | 9.45±1.75 ** | 13.0±1.70 ** |
| NC-IM+AC0.2 | 22.5±1.81 ++ | 20.3±2.20 ++ |
| NC-IM+AC1 | 19.8±1.83 *,++ | 19.8±2.21 *,++ |
| (D) CZ groups | | |
| Groups | Percentage of entries into open arms | Percentage of time spent on open arms |
| Control | 21.7±2.14 | 22.0±2.35 |
| CZ0.1 | 22.3±2.16 | 22.3±2.56 |
| CZ1 | 22.7±2.24 | 22.7±2.69 |
| CZ5 | 22.9±2.28 | 22.8±2.70 |
| NC | 9.16±1.14 ** | 13.5±1.61 ** |
| NC+CZ0.1 | 10.5±1.59 **,+ | 15.0±1.72 ** |
| NC+CZ1 | 21.6±1.73 ++ | 20.6±2.12 ++ |
| NC+CZ5 | 20.6±1.83 ++ | 20.3±2.13 ++ |
| IM | 11.7±1.23 ** | 15.2±1.59 ** |
| IM+CZ0.1 | 13.0±1.33 **,+ | 16.8±1.82 ** |
| IM+CZ1 | 22.2±1.81 ++ | 21.4±2.42 ++ |
| IM+CZ5 | 21.3±1.86 ++ | 21.0±2.42 ++ |
| NC-IM | 9.18±1.03 **,$$ | 12.3±1.61 **,$$ |
| NC-IM+CZ0.1 | 9.85±1.76 ** | 13.1±1.73 ** |
| NC-IM+CZ1 | 22.9±1.87 ++ | 20.4±2.22 ++ |
| NC-IM+CZ5 | 20.3±1.88 ++ | 20.2±2.23 ++ |

The parameter values of the EPM test at the 2 h time point after the last NC (0.8 mg/kg, s.c.) and/or IM (10 min) treatment are shown as means ± SD (n=10) for each HDAC inhibitor (SB or VA), CB1 agonist (AC) or TRPV1 antagonist (CZ) co-treatment group (with each i.p. dose (mg/kg)), and statistical significance in post-hoc tests is denoted using the symbols as defined below. (A) SB (50, 100 and 200 mg/kg) co-treatment groups (SB groups); (B) VA (200, 300 and 400 mg/kg) co-treatment groups (VA groups); (C) AC (0.05, 0.2 and 1 mg/kg) co-treatment groups (AC groups); (D) CZ (0.1, 1 and 5 mg/kg) co-treatment groups (CZ groups). * (p<0.05), ** (p<0.01): significant attenuation as compared to the control group; + (p<0.05), ++ (p<0.01): significant increase as compared to the NC, IM or NC-IM group without any co-treatments; $$ (p<0.01): significant attenuation as compared to the IM group without any co-treatments.

**TABLE S2.** Data for the mitigating effects of the HDAC inhibitors, TRPV1 agonist or CB1 antagonist against working memory impairment-like behaviors, which are summarized and depicted in FIGURE 2.

| (A) SB groups | |
| --- | --- |
| Groups | SAP rate |
| Control | 63.6±8.60 |
| SB50 | 64.7±8.59 |
| SB100 | 65.0±8.65 |
| SB200 | 65.3±8.76 |
| NC | 53.2±7.21 ** |
| NC+SB50 | 58.4±7.53 |
| NC+SB100 | 63.1±7.87 ++ |
| NC+SB200 | 61.5±7.97 + |
| IM | 53.5±6.50 ** |
| IM+SB50 | 59.1±8.20 |
| IM+SB100 | 64.8±8.41 ++ |
| IM+SB200 | 64.0±8.60 ++ |
| NC-IM | 44.7±5.43 **,@@,$$ |
| NC-IM+SB50 | 50.2±6.20 **,+ |
| NC-IM+SB100 | 59.4±6.52 ++ |
| NC-IM+SB200 | 58.0±6.78 ++ |
| (B) VA groups | |
| Groups | SAP rate |
| Control | 63.6±8.60 |
| VA200 | 64.8±8.60 |
| VA300 | 65.1±8.69 |
| VA400 | 65.4±8.74 |
| NC | 53.2±7.21 ** |
| NC+VA200 | 60.1±7.46 + |
| NC+VA300 | 63.2±7.60 ++ |
| NC+VA400 | 61.7±7.88 + |
| IM | 53.5±6.50 ** |
| IM+VA200 | 60.4±8.02 + |
| IM+VA300 | 65.3±8.32 ++ |
| IM+VA400 | 64.0±8.50 ++ |
| NC-IM | 44.7±5.43 **,@@,$$ |
| NC-IM+VA200 | 51.0±5.97 **,+ |
| NC-IM+VA300 | 60.3±6.41 ++ |
| NC-IM+VA400 | 58.5±6.57 ++ |
| (C) OL groups | |
| Groups | SAP rate |
| Control | 63.6±8.60 |
| OL0.1 | 63.9±8.81 |
| OL1 | 64.5±8.91 |
| OL2.5 | 64.9±9.03 |
| NC | 53.2±7.21 ** |
| NC+OL0.1 | 54.7±7.33 * |
| NC+OL1 | 62.9±7.60 ++ |
| NC+OL2.5 | 60.2±7.68 + |
| IM | 53.5±6.50 ** |
| IM+OL0.1 | 54.7±6.84 * |
| IM+OL1 | 64.1±7.12 ++ |
| IM+OL2.5 | 60.3±7.31 + |
| NC-IM | 44.7±5.43 **,@@,$$ |
| NC-IM+OL0.1 | 48.5±5.68 ** |
| NC-IM+OL1 | 58.8±6.04 ++ |
| NC-IM+OL2.5 | 55.2±6.30 *,++ |
| (D) SR groups | |
| Groups | SAP rate |
| Control | 63.6±8.60 |
| SR0.5 | 63.8±8.74 |
| SR1 | 64.4±8.90 |
| SR2 | 64.9±8.97 |
| NC | 53.2±7.21 ** |
| NC+SR0.5 | 54.8±7.27 * |
| NC+SR1 | 63.0±7.52 ++ |
| NC+SR2 | 60.3±7.62 + |
| IM | 53.5±6.50 ** |
| IM+SR0.5 | 54.9±6.72 * |
| IM+SR1 | 64.2±7.08 ++ |
| IM+SR2 | 60.4±7.21 + |
| NC-IM | 44.7±5.43 **,@@,$$ |
| NC-IM+SR0.5 | 49.0±5.92 ** |
| NC-IM+SR1 | 59.1±5.96 ++ |
| NC-IM+SR2 | 57.9±6.25 ++ |

The parameter values of the Y maze test (SAP rates) at the 2 h time point after the last NC (0.8 mg/kg, s.c.) and/or IM (10 min) treatment are shown as means ± SD (n=10) for each HDAC inhibitor (SB or VA), TRPV1 agonist (OL) or CB1 antagonist (SR) co-treatment group (with each i.p. dose (mg/kg)), and statistical significance in post-hoc tests is denoted using the symbols as defined below. (A) SB (50, 100 and 200 mg/kg) co-treatment groups (SB groups); (B) VA (200, 300 and 400 mg/kg) co-treatment groups (VA groups); (C) OL (0.1, 1 and 2.5 mg/kg) co-treatment groups (OL groups); (D) SR (0.5, 1 and 2 mg/kg) co-treatment groups (SR groups). * (p<0.05), ** (p<0.01): significant attenuation as compared to the control group; + (p<0.05), ++ (p<0.01): significant increase as compared to the NC, IM or NC-IM group without any co-treatments; @@ (p<0.01): significant attenuation as compared to the NC group without any co-treatments; $$ (p<0.01): significant attenuation as compared to the IM group without any co-treatments.

**TABLE S3.** Data for the interacting effects between the CB1 antagonist and mitigating (anxiolytic-like) drug (i.e., HDAC inhibitor, CB1 agonist or TRPV1 antagonist) against anxiety-like behavioral alterations caused by NC and/or IM, which are summarized and depicted in FIGURE 3.

| (A) SB+SR groups | | |
| --- | --- | --- |
| Groups | Percentage of entries into open arms | Percentage of time spent on open arms |
| Control | 21.7±2.14 | 22.0±2.35 |
| SB | 22.7±2.31 | 22.7±2.62 |
| SB+SR0.5 | 22.4±2.00 | 22.4±2.67 |
| SB+SR1 | 21.9±2.13 | 22.1±2.76 |
| SB+SR2 | 22.2±2.16 | 22.3±2.83 |
| NC | 9.16±1.14 ** | 13.5±1.61 ** |
| NC+SB | 21.0±1.78 ++ | 20.5±2.16 ++ |
| NC+SB+SR0.5 | 20.5±1.80 ++ | 20.2±2.22 ++ |
| NC+SB+SR1 | 9.91±1.05 **,## | 14.6±1.60 **,## |
| NC+SB+SR2 | 10.3±1.16 **,+,## | 16.7±1.78 **,++,## |
| IM | 11.7±1.23 ** | 15.2±1.59 ** |
| IM+SB | 21.5±1.54 ++ | 21.2±2.17 ++ |
| IM+SB+SR0.5 | 20.3±1.61 ++ | 20.2±2.18 ++ |
| IM+SB+SR1 | 11.9±1.25 **,## | 16.1±1.75 **,## |
| IM+SB+SR2 | 12.4±1.26 **,## | 16.4±1.79 **,## |
| NC-IM | 9.18±1.03 **,$$ | 12.3±1.61 **,$$ |
| NC-IM+SB | 21.4±1.30 ++ | 20.8±2.21 ++ |
| NC-IM+SB+SR0.5 | 20.8±1.52 ++ | 20.5±2.22 ++ |
| NC-IM+SB+SR1 | 9.84±1.03 **,## | 13.2±1.56 **,## |
| NC-IM+SB+SR2 | 10.6±1.08 **,++,## | 15.2±1.60 **,++,## |
| (B) VA+SR groups | | |
| Groups | Percentage of entries into open arms | Percentage of time spent on open arms |
| Control | 21.7±2.14 | 22.0±2.35 |
| VA | 22.5±2.25 | 22.7±2.62 |
| VA+SR0.5 | 22.3±2.25 | 22.4±2.68 |
| VA+SR1 | 22.0±2.30 | 22.0±2.75 |
| VA+SR2 | 22.2±2.33 | 22.3±2.81 |
| NC | 9.16±1.14 ** | 13.5±1.61 ** |
| NC+VA | 21.3±1.73 ++ | 21.0±2.21 ++ |
| NC+VA+SR0.5 | 20.5±1.73 ++ | 20.7±2.28 ++ |
| NC+VA+SR1 | 9.73±1.16 **,## | 14.6±1.58 **,## |
| NC+VA+SR2 | 10.6±1.17 **,+,## | 17.0±1.81 **,++,## |
| IM | 11.7±1.23 ** | 15.2±1.59 ** |
| IM+VA | 22.0±1.86 ++ | 21.8±2.22 ++ |
| IM+VA+SR0.5 | 20.7±1.87 ++ | 20.7±2.23 ++ |
| IM+VA+SR1 | 12.1±1.16 **,## | 16.2±1.77 **,## |
| IM+VA+SR2 | 12.5±1.18 **,## | 16.4±1.81 **,## |
| NC-IM | 9.18±1.03 **,$$ | 12.3±1.61 **,$$ |
| NC-IM+VA | 21.4±1.45 ++ | 20.9±2.21 ++ |
| NC-IM+VA+SR0.5 | 20.6±1.50 ++ | 20.7±2.23 ++ |
| NC-IM+VA+SR1 | 9.87±1.07 **,## | 13.3±1.56 **,## |
| NC-IM+VA+SR2 | 10.8±1.15 **,++,## | 15.3±1.63 **,++,## |
| (C) AC+SR groups | | |
| Groups | Percentage of entries into open arms | Percentage of time spent on open arms |
| Control | 21.7±2.14 | 22.0±2.35 |
| AC | 22.6±2.06 | 22.7±2.68 |
| AC+SR0.5 | 22.2±2.06 | 22.4±2.73 |
| AC+SR1 | 21.8±2.13 | 22.1±2.80 |
| AC+SR2 | 22.0±2.24 | 22.4±2.87 |
| NC | 9.16±1.14 ** | 13.5±1.61 ** |
| NC+AC | 21.4±1.73 ++ | 20.4±2.08 ++ |
| NC+AC+SR0.5 | 19.7±1.76 *,++,# | 18.3±2.08 **,++,# |
| NC+AC+SR1 | 9.16±1.06 **,## | 14.0±1.53 **,## |
| NC+AC+SR2 | 9.72±1.54 **,## | 14.7±1.70 **,## |
| IM | 11.7±1.23 ** | 15.2±1.59 ** |
| IM+AC | 21.7±1.78 ++ | 21.2±2.37 ++ |
| IM+AC+SR0.5 | 19.9±1.81 ++,# | 17.5±2.09 **,+,## |
| IM+AC+SR1 | 11.6±1.35 **,## | 15.5±1.74 **,## |
| IM+AC+SR2 | 12.2±1.49 **,## | 15.8±1.81 **,## |
| NC-IM | 9.18±1.03 **,$$ | 12.3±1.61 **,$$ |
| NC-IM+AC | 22.5±1.81 ++ | 20.3±2.20 ++ |
| NC-IM+AC+SR0.5 | 21.1±1.87 ++ | 17.6±1.90 **,++,## |
| NC-IM+AC+SR1 | 9.47±1.16 **,## | 12.6±1.54 **,## |
| NC-IM+AC+SR2 | 10.3±1.20 **,+,## | 14.5±1.60 **,++,## |
| (D) CZ+SR groups | | |
| Groups | Percentage of entries into open arms | Percentage of time spent on open arms |
| Control | 21.7±2.14 | 22.0±2.35 |
| CZ | 22.7±2.24 | 22.7±2.69 |
| CZ+SR0.5 | 22.4±2.28 | 22.5±2.75 |
| CZ+SR1 | 22.1±2.31 | 22.3±2.82 |
| CZ+SR2 | 22.3±2.32 | 22.5±2.89 |
| NC | 9.16±1.14 ** | 13.5±1.61 ** |
| NC+CZ | 21.6±1.73 ++ | 20.6±2.12 ++ |
| NC+CZ+SR0.5 | 20.3±1.76 ++ | 18.5±2.12 **,++,# |
| NC+CZ+SR1 | 9.53±1.05 **,## | 14.2±1.54 **,## |
| NC+CZ+SR2 | 9.91±1.53 **,## | 14.7±1.71 **,## |
| IM | 11.7±1.23 ** | 15.2±1.59 ** |
| IM+CZ | 22.2±1.81 ++ | 21.4±2.42 ++ |
| IM+CZ+SR0.5 | 20.8±1.85 ++ | 19.1±2.15 *,++,# |
| IM+CZ+SR1 | 12.2±1.34 **,## | 15.9±1.80 **,## |
| IM+CZ+SR2 | 12.6±1.44 **,## | 16.1±1.84 **,## |
| NC-IM | 9.18±1.03 **,$$ | 12.3±1.61 **,$$ |
| NC-IM+CZ | 22.9±1.87 ++ | 20.4±2.22 ++ |
| NC-IM+CZ+SR0.5 | 21.8±1.90 ++ | 17.7±1.97 **,++,## |
| NC-IM+CZ+SR1 | 9.93±1.16 **,## | 12.7±1.59 **,## |
| NC-IM+CZ+SR2 | 10.7±1.18 **,++,## | 14.6±1.65 **,++,## |

The parameter values of the EPM test at the 2 h time point after the last NC (0.8 mg/kg, s.c.) or IM (10 min) treatment are shown as means ± SD (n=10) for each mitigating drug plus CB1 antagonist group (with each i.p. dose (mg/kg)), and statistical significance in post-hoc tests is denoted using the symbols as defined below. (A) SB (100 mg/kg) plus SR (0.5, 1 and 2 mg/kg) groups (SB+SR groups); (B) VA (300 mg/kg) plus SR (0.5, 1 and 2 mg/kg) groups (VA+SR groups); (C) AC (0.2 mg/kg) plus SR (0.5, 1 and 2 mg/kg) groups (AC+SR groups); (D) CZ (1 mg/kg) plus SR (0.5, 1 and 2 mg/kg) groups (CZ+SR groups). * (p<0.05), ** (p<0.01): significant attenuation as compared to the control group; + (p<0.05), ++ (p<0.01): significant increase as compared to the NC, IM or NC-IM group without any co-treatments; $$ (p<0.01): significant attenuation as compared to the IM group without any co-treatments; # (p<0.05), ## (p<0.01): significant attenuation as compared to the NC, IM or NC-IM group co-treated with the efficacious HDAC inhibitor, CB1 agonist or TRPV1 antagonist.

**TABLE S4.** Data for the counteraction caused by the TRPV1 antagonist against attenuating effects of the CB1 antagonist on HDAC inhibitor- or CB1 agonist-induced anxiolytic-like behavioral alterations in the NC and/or IM treatment groups, which are depicted in FIGURE 4.

| (A) SB+SR+CZ groups | | |
| --- | --- | --- |
| Groups | Percentage of entries into open arms | Percentage of time spent on open arms |
| Control | 21.7±2.14 | 22.0±2.35 |
| SB | 22.7±2.31 | 22.7±2.62 |
| SB+SR | 21.9±2.13 | 22.1±2.76 |
| SB+SR+CZ | 22.3±2.19 | 22.3±2.78 |
| NC | 9.16±1.14 ** | 13.5±1.61 ** |
| NC+SB | 21.0±1.78 ++ | 20.5±2.16 ++ |
| NC+SB+SR | 9.91±1.05 **,## | 14.6±1.60 **,## |
| NC+SB+SR+CZ | 21.2±1.88 ++,&& | 19.9±2.09 ++,&& |
| IM | 11.7±1.23 ** | 15.2±1.59 ** |
| IM+SB | 21.5±1.54 ++ | 21.2±2.17 ++ |
| IM+SB+SR | 11.9±1.25 **,## | 16.1±1.75 **,## |
| IM+SB+SR+CZ | 21.4±1.56 ++,&& | 20.6±2.04 ++,&& |
| NC-IM | 9.18±1.03 **,$$ | 12.3±1.61 **,$$ |
| NC-IM+SB | 21.4±1.30 ++ | 20.8±2.21 ++ |
| NC-IM+SB+SR | 9.84±1.03 **,## | 13.2±1.56 **,## |
| NC-IM+SB+SR+CZ | 21.3±1.32 ++,&& | 20.0±1.96 ++,&& |
| (B) VA+SR+CZ groups | | |
| Groups | Percentage of entries into open arms | Percentage of time spent on open arms |
| Control | 21.7±2.14 | 22.0±2.35 |
| VA | 22.5±2.25 | 22.7±2.62 |
| VA+SR | 22.0±2.30 | 22.0±2.75 |
| VA+SR+CZ | 22.2±2.46 | 22.2±2.79 |
| NC | 9.16±1.14 ** | 13.5±1.61 ** |
| NC+VA | 21.3±1.73 ++ | 21.0±2.21 ++ |
| NC+VA+SR | 9.73±1.16 **,## | 14.6±1.58 **,## |
| NC+VA+SR+CZ | 21.4±1.56 ++,&& | 19.9±2.07 ++,&& |
| IM | 11.7±1.23 ** | 15.2±1.59 ** |
| IM+VA | 22.0±1.86 ++ | 21.8±2.22 ++ |
| IM+VA+SR | 12.1±1.16 **,## | 16.2±1.77 **,## |
| IM+VA+SR+CZ | 21.9±1.32 ++,&& | 20.7±1.98 ++,&& |
| NC-IM | 9.18±1.03 **,$$ | 12.3±1.61 **,$$ |
| NC-IM+VA | 21.4±1.45 ++ | 20.9±2.21 ++ |
| NC-IM+VA+SR | 9.87±1.07 **,## | 13.3±1.56 **,## |
| NC-IM+VA+SR+CZ | 21.6±1.45 ++,&& | 20.2±2.06 ++,&& |
| (C) AC+SR+CZ groups | | |
| Groups | Percentage of entries into open arms | Percentage of time spent on open arms |
| Control | 21.7±2.14 | 22.0±2.35 |
| AC | 22.6±2.06 | 22.7±2.68 |
| AC+SR | 21.8±2.13 | 22.1±2.80 |
| AC+SR+CZ | 22.1±2.27 | 22.3±2.82 |
| NC | 9.16±1.14 ** | 13.5±1.61 ** |
| NC+AC | 21.4±1.73 ++ | 20.4±2.08 ++ |
| NC+AC+SR | 9.16±1.06 **,## | 14.0±1.53 **,## |
| NC+AC+SR+CZ | 20.5±0.995 ++,&& | 19.5±2.04 ++,&& |
| IM | 11.7±1.23 ** | 15.2±1.59 ** |
| IM+AC | 21.7±1.78 ++ | 21.2±2.37 ++ |
| IM+AC+SR | 11.6±1.35 **,## | 15.5±1.74 **,## |
| IM+AC+SR+CZ | 21.0±1.03 ++,&& | 19.6±2.08 ++,&& |
| NC-IM | 9.18±1.03 **,$$ | 12.3±1.61 **,$$ |
| NC-IM+AC | 22.5±1.81 ++ | 20.3±2.20 ++ |
| NC-IM+AC+SR | 9.47±1.16 **,## | 12.6±1.54 **,## |
| NC-IM+AC+SR+CZ | 21.9±1.05 ++,&& | 19.7±2.19 ++,&& |

The parameter values of the EPM test at the 2 h time point after the last NC (0.8 mg/kg, s.c.) and/or IM (10 min) treatment are shown as means ± SD (n=10) for each anxiolytic-like drug (HDAC inhibitor or CB1 agonist) plus CB1 antagonist co-treatment group with or without additional TRPV1 antagonist (with each i.p. dose (mg/kg)), and statistical significance in post-hoc tests is denoted using the same symbols as FIGURE 4. (A) SB (100 mg/kg) plus SR (1 mg/kg) co-treatment groups with (or without) additional CZ (1 mg/kg) (SB+SR+CZ groups); (B) VA (300 mg/kg) plus SR (1 mg/kg) co-treatment groups with (or without) additional CZ (1 mg/kg) (VA+SR+CZ groups); (C) AC (0.2 mg/kg) plus SR (1 mg/kg) co-treatment groups with (or without) additional CZ (1 mg/kg) (AC+SR+CZ groups).

**TABLE S5.** Data for the interacting effects between the TRPV1 antagonist and mitigating (working memory improving-like) drug (i.e., HDAC inhibitor, TRPV1 agonist or CB1 antagonist) against working memory impairment-like behavioral alterations caused by NC and/or IM, which are summarized and depicted in FIGURE 5.

| (A) SB+CZ groups | |
| --- | --- |
| Groups | SAP rate |
| Control | 63.6±8.60 |
| SB | 65.0±8.65 |
| SB+CZ0.1 | 65.0±8.68 |
| SB+CZ1 | 64.9±8.72 |
| SB+CZ5 | 64.8±8.75 |
| NC | 53.2±7.21 ** |
| NC+SB | 63.1±7.87 ++ |
| NC+SB+CZ0.1 | 61.7±8.06 + |
| NC+SB+CZ1 | 52.7±8.08 **,## |
| NC+SB+CZ5 | 54.9±8.15 *,# |
| IM | 53.5±6.50 ** |
| IM+SB | 64.8±8.41 ++ |
| IM+SB+CZ0.1 | 63.5±8.54 ++ |
| IM+SB+CZ1 | 52.3±8.57 **.## |
| IM+SB+CZ5 | 55.2±8.63 *,# |
| NC-IM | 44.7±5.43 **,@@,$$ |
| NC-IM+SB | 59.4±6.52 ++ |
| NC-IM+SB+CZ0.1 | 55.6±6.56 *,++ |
| NC-IM+SB+CZ1 | 48.7±6.65 **.## |
| NC-IM+SB+CZ5 | 49.4±6.69 **.## |
| (B) VA+CZ groups | |
| Groups | SAP rate |
| Control | 63.6±8.60 |
| VA | 65.1±8.69 |
| VA+CZ0.1 | 65.1±8.71 |
| VA+CZ1 | 64.9±8.78 |
| VA+CZ5 | 64.9±8.79 |
| NC | 53.2±7.21 ** |
| NC+VA | 63.2±7.60 ++ |
| NC+VA+CZ0.1 | 61.9±8.01 + |
| NC+VA+CZ1 | 52.8±8.02 **.## |
| NC+VA+CZ5 | 55.0±8.12 *,# |
| IM | 53.5±6.50 ** |
| IM+VA | 65.3±8.32 ++ |
| IM+VA+CZ0.1 | 63.7±8.39 ++ |
| IM+VA+CZ1 | 52.4±8.46 **.## |
| IM+VA+CZ5 | 57.1±8.62 # |
| NC-IM | 44.7±5.43 **,@@,$$ |
| NC-IM+VA | 60.3±6.41 ++ |
| NC-IM+VA+CZ0.1 | 55.6±6.53 *,++ |
| NC-IM+VA+CZ1 | 48.8±6.56 **.## |
| NC-IM+VA+CZ5 | 49.4±6.67 **.## |
| (C) OL+CZ groups | |
| Groups | SAP rate |
| Control | 63.6±8.60 |
| OL | 64.5±8.91 |
| OL+CZ0.1 | 64.5±8.92 |
| OL+CZ1 | 64.4±8.95 |
| OL+CZ5 | 64.4±8.96 |
| NC | 53.2±7.21 ** |
| NC+OL | 62.9±7.60 ++ |
| NC+OL+CZ0.1 | 61.5±7.98 + |
| NC+OL+CZ1 | 52.7±7.99 **,## |
| NC+OL+CZ5 | 54.8±8.09 *,# |
| IM | 53.5±6.50 ** |
| IM+OL | 64.1±7.12 ++ |
| IM+OL+CZ0.1 | 63.4±8.35 ++ |
| IM+OL+CZ1 | 52.1±8.43 **,## |
| IM+OL+CZ5 | 55.2±8.57 *,# |
| NC-IM | 44.7±5.43 **,@@,$$ |
| NC-IM+OL | 58.8±6.04 ++ |
| NC-IM+OL+CZ0.1 | 55.3±6.37 *,++ |
| NC-IM+OL+CZ1 | 48.5±6.49 **,## |
| NC-IM+OL+CZ5 | 49.2±6.61 **,## |
| (D) SR+CZ groups | |
| Groups | SAP rate |
| Control | 63.6±8.60 |
| SR | 64.4±8.90 |
| SR+CZ0.1 | 64.3±8.91 |
| SR+CZ1 | 64.3±8.92 |
| SR+CZ5 | 64.3±8.92 |
| NC | 53.2±7.21 ** |
| NC+SR | 63.0±7.52 ++ |
| NC+SR+CZ0.1 | 61.5±7.88 + |
| NC+SR+CZ1 | 52.8±7.98 **,## |
| NC+SR+CZ5 | 54.9±8.03 *,# |
| IM | 53.5±6.50 ** |
| IM+SR | 64.2±7.08 ++ |
| IM+SR+CZ0.1 | 63.4±8.32 ++ |
| IM+SR+CZ1 | 52.3±8.41 **,## |
| IM+SR+CZ5 | 55.2±8.54 *,# |
| NC-IM | 44.7±5.43 **,@@,$$ |
| NC-IM+SR | 59.1±5.96 ++ |
| NC-IM+SR+CZ0.1 | 55.5±6.23 *,++ |
| NC-IM+SR+CZ1 | 48.7±6.46 **,## |
| NC-IM+SR+CZ5 | 49.3±6.56 **,## |

The parameter values of the Y maze test at the 2 h time point after the last NC (0.8 mg/kg, s.c.) or IM (10 min) treatment are shown as means ± SD (n=10) for each mitigating drug plus TRPV1 antagonist group (with each i.p. dose (mg/kg)), and statistical significance in post-hoc tests is denoted using the symbols as defined below. (A) SB (100 mg/kg) plus CZ (0.1, 1 and 5 mg/kg) groups (SB+CZ groups); (B) VA (300 mg/kg) plus CZ (0.1, 1 and 5 mg/kg) groups (VA+CZ groups); (C) OL (1 mg/kg) plus CZ (0.1, 1 and 5 mg/kg) groups (OL+CZ groups); (D) SR (1 mg/kg) plus CZ (0.1, 1 and 5 mg/kg) groups (SR+CZ groups). * (p<0.05), ** (p<0.01): significant attenuation as compared to the control group; + (p<0.05), ++ (p<0.01): significant increase as compared to the NC, IM or NC-IM group without any co-treatments; @@ (p<0.01): significant attenuation as compared to the NC group without any co-treatments; $$ (p<0.01): significant attenuation as compared to the IM group without any co-treatments; # (p<0.05), ## (p<0.01): significant attenuation as compared to the NC, IM or NC-IM group co-treated with the efficacious HDAC inhibitor, TRPV1 agonist or CB1 antagonist.

**TABLE S6.** Data for the counteraction caused by the CB1 antagonist against attenuating effects of the TRPV1 antagonist on HDAC inhibitor- or TRPV1 agonist-induced working memory improving-like behavioral alterations in the NC and/or IM treatment groups, which are depicted in FIGURE 6.

| (A) SB+CZ+SR groups | |
| --- | --- |
| Groups | SAP rate |
| Control | 63.6±8.60 |
| SB | 65.0±8.65 |
| SB+CZ | 64.9±8.72 |
| SB+CZ+SR | 66.1± 6.11 |
| NC | 53.2±7.21 ** |
| NC+SB | 63.1±7.87 ++ |
| NC+SB+CZ | 52.7±8.08 **,## |
| NC+SB+CZ+SR | 69.3± 6.07 ++,&& |
| IM | 53.5±6.50 ** |
| IM+SB | 64.8±8.41 ++ |
| IM+SB+CZ | 52.3±8.57 **.## |
| IM+SB+CZ+SR | 70.6± 6.14 ++,&& |
| NC-IM | 44.7±5.43 **,@@,$$ |
| NC-IM+SB | 59.4±6.52 ++ |
| NC-IM+SB+CZ | 48.7±6.65 **.## |
| NC-IM+SB+CZ+SR | 66.4±6.58 ++,&& |
| (B) VA+CZ+SR groups | |
| Groups | SAP rate |
| Control | 63.6±8.60 |
| VA | 65.1±8.69 |
| VA+CZ | 64.9±8.78 |
| VA+CZ+SR | 66.2± 6.14 |
| NC | 53.2±7.21 ** |
| NC+VA | 63.2±7.60 ++ |
| NC+VA+CZ | 52.8±8.02 **.## |
| NC+VA+CZ+SR | 69.8± 6.52 ++,&& |
| IM | 53.5±6.50 ** |
| IM+VA | 65.3±8.32 ++ |
| IM+VA+CZ | 52.4±8.46 **.## |
| IM+VA+CZ+SR | 70.4± 6.57 ++,&& |
| NC-IM | 44.7±5.43 **,@@,$$ |
| NC-IM+VA | 60.3±6.41 ++ |
| NC-IM+VA+CZ | 48.8±6.56 **.## |
| NC-IM+VA+CZ+SR | 66.5± 6.43 ++,&& |
| (C) OL+CZ+SR groups | |
| Groups | SAP rate |
| Control | 63.6±8.60 |
| OL | 64.5±8.91 |
| OL+CZ | 64.4±8.95 |
| OL+CZ+SR | 65.8± 6.35 |
| NC | 53.2±7.21 ** |
| NC+OL | 62.9±7.60 ++ |
| NC+OL+CZ | 52.7±7.99 **,## |
| NC+OL+CZ+SR | 69.7± 6.21 ++,&& |
| IM | 53.5±6.50 ** |
| IM+OL | 64.1±7.12 ++ |
| IM+OL+CZ | 52.1±8.43 **,## |
| IM+OL+CZ+SR | 70.1±6.19 ++,&& |
| NC-IM | 44.7±5.43 **,@@,$$ |
| NC-IM+OL | 58.8±6.04 ++ |
| NC-IM+OL+CZ | 48.5±6.49 **,## |
| NC-IM+OL+CZ+SR | 66.3±6.47 ++,&& |

The parameter values of the Y maze test at the 2 h time point after the last NC (0.8 mg/kg, s.c.) and/or IM (10 min) treatment are shown as means ± SD (n=10) for each working memory improving-like drug (HDAC inhibitor or TRPV1 agonist) plus TRPV1 antagonist co-treatment group with or without additional CB1 antagonist (with each i.p. dose (mg/kg)), and statistical significance in post-hoc tests is denoted using the same symbols as FIGURE 6. (A) SB (100 mg/kg) plus CZ (1 mg/kg) co-treatment groups with (or without) additional SR (1 mg/kg) (SB+CZ+SR groups); (B) VA (300 mg/kg) plus CZ (1 mg/kg) co-treatment groups with (or without) additional SR (1 mg/kg) (VA+CZ+SR groups); (C) OL (1 mg/kg) plus CZ (1 mg/kg) co-treatment groups with (or without) additional SR (1 mg/kg) (OL+CZ+SR groups).

**TABLE S7.** Results of two- or three-way analysis of variance (ANOVA) for the parameter values of anxiety-related behavioral plasticity in the EPM test (percentages of entries into open arms and time spent on open arms).

(A) Percentage of entries into open arms

| Main and interacting factors | ANOVA results | Relevant figures and tables |
| --- | --- | --- |
| NC | F(1, 36)=268.59, P=2.84×10^-18^ (P<0.001) | Figure 1 and Table S1 |
| IM | F(1, 36)=116.92, P=7.39×10^-13^ (P<0.001) | Figure 1 and Table S1 |
| NC×IM | F(1, 36)=118.07, P=6.45×10^-13^ (P<0.001) | Figure 1 and Table S1 |
| NC and/or IM × SB | F(9, 144)=21.73, P=6.85×10^-23^ (P<0.001) | Figure 1 and Table S1 |
| NC and/or IM × VA | F(9, 144)=22.12, P=3.31×10^-23^ (P<0.001) | Figure 1 and Table S1 |
| NC and/or IM × AC | F(9, 144)=30.53, P=2.97×10^-29^ (P<0.001) | Figure 1 and Table S1 |
| NC and/or IM × CZ | F(9, 144)=29.13, P=2.53×10^-28^ (P<0.001) | Figure 1 and Table S1 |
| NC and/or IM × SB × SR | F(9, 288)=15.96, P=3.60×10^-21^ (P<0.001) | Figure 3 and Table S3 |
| NC and/or IM × VA × SR | F(9, 288)=15.63, P=9.24×10^-21^ (P<0.001) | Figure 3 and Table S3 |
| NC and/or IM × AC × SR | F(9, 288)=16.89, P=2.55×10^-22^ (P<0.001) | Figure 3 and Table S3 |
| NC and/or IM × CZ × SR | F(9, 288)=17.16, P=1.20×10^-22^ (P<0.001) | Figure 3 and Table S3 |
| SB plus SR × CZ | F(1, 144)=5.54, P=0.0199 (P<0.05) | Figure 4 and Table S4 |
| VA plus SR × CZ | F(1, 144)=3.96, P=0.0484 (P<0.05) | Figure 4 and Table S4 |
| AC plus SR × CZ | F(1, 144)=4.18, P=0.0427 (P<0.05) | Figure 4 and Table S4 |
| NC and/or IM × SB plus SR × CZ | F(3, 144)=0.48, P=0.697 | Figure 4 and Table S4 |
| NC and/or IM × VA plus SR × CZ | F(3, 144)=0.38, P=0.769 | Figure 4 and Table S4 |
| NC and/or IM × AC plus SR × CZ | F(3, 144)=0.10, P=0.962 | Figure 4 and Table S4 |

(B) Percentage of time spent on open arms

| Main and interacting factors | ANOVA results | Relevant figures and tables |
| --- | --- | --- |
| NC | F(1, 36)=96.98, P=9.34×10^-12^ (P<0.001) | Figure 1 and Table S1 |
| IM | F(1, 36)=48.32, P=3.80×10^-8^ (P<0.001) | Figure 1 and Table S1 |
| NC×IM | F(1, 36)=23.68, P=2.26×10^-5^ (P<0.001) | Figure 1 and Table S1 |
| NC and/or IM × SB | F(9, 144)=5.42, P=2.16×10^-6^ (P<0.001) | Figure 1 and Table S1 |
| NC and/or IM × VA | F(9, 144)=5.73, P=8.85×10^-7^ (P<0.001) | Figure 1 and Table S1 |
| NC and/or IM × AC | F(9, 144)=6.58, P=7.70×10^-8^ (P<0.001) | Figure 1 and Table S1 |
| NC and/or IM × CZ | F(9, 144)=6.68, P=5.78×10^-8^ (P<0.001) | Figure 1 and Table S1 |
| NC and/or IM × SB × SR | F(9, 288)=3.06, P=0.00162 (P<0.01) | Figure 3 and Table S3 |
| NC and/or IM × VA × SR | F(9, 288)=3.21, P=0.00101 (P<0.01) | Figure 3 and Table S3 |
| NC and/or IM × AC × SR | F(9, 288)=2.85, P=0.00308 (P<0.01) | Figure 3 and Table S3 |
| NC and/or IM × CZ × SR | F(9, 288)=2.76, P=0.00410 (P<0.01) | Figure 3 and Table S3 |
| SB plus SR × CZ | F(1, 144)=3.98, P=0.0480 (P<0.05) | Figure 4 and Table S4 |
| VA plus SR × CZ | F(1, 144)=4.07, P=0.0454 (P<0.05) | Figure 4 and Table S4 |
| AC plus SR × CZ | F(1, 144)=3.92, P=0.0496 (P<0.05) | Figure 4 and Table S4 |
| NC and/or IM × SB plus SR × CZ | F(3, 144)=0.16, P=0.920 | Figure 4 and Table S4 |
| NC and/or IM × VA plus SR × CZ | F(3, 144)=0.15, P=0.929 | Figure 4 and Table S4 |
| NC and/or IM × AC plus SR × CZ | F(3, 144)=0.25, P=0.862 | Figure 4 and Table S4 |

**TABLE S8.** Results of two- or three-way analysis of variance (ANOVA) for the parameter values of working memory impairment-related behavioral plasticity in the Y maze test (SAP rate).

| Main and interacting factors | ANOVA results  (SAP rate) | Relevant figures and tables |
| --- | --- | --- |
| NC | F(1, 36)=18.72, P=1.15×10^-4^ (P<0.001) | Figure 2 and Table S2 |
| IM | F(1, 36)=17.62, P=1.69×10^-4^ (P<0.001) | Figure 2 and Table S2 |
| NC×IM | F(1, 36)= 0.13, P= 0.718 | Figure 2 and Table S2 |
| SB | F(3, 144)=12.45, P=2.75×10^-7^ (P<0.001) | Figure 2 and Table S2 |
| VA | F(3, 144)=13.32, P=1.01×10^-7^ (P<0.001) | Figure 2 and Table S2 |
| OL | F(3, 144)=12.36, P=3.07×10^-7^ (P<0.001) | Figure 2 and Table S2 |
| SR | F(3, 144)=13.29, P=1.05×10^-7^ (P<0.001) | Figure 2 and Table S2 |
| NC and/or IM × SB | F(9, 144)=1.19, P= 0.304 | Figure 2 and Table S2 |
| NC and/or IM × VA | F(9, 144)=1.35, P= 0.216 | Figure 2 and Table S2 |
| NC and/or IM × OL | F(9, 144)=1.21, P=0.296 | Figure 2 and Table S2 |
| NC and/or IM × SR | F(9, 144)=1.46, P=0.170 | Figure 2 and Table S2 |
| SB × CZ | F(3, 288)=4.74, P=0.00304 (P<0.01) | Figure 5 and Table S5 |
| VA × CZ | F(3, 288)=4.92, P=0.00240 (P<0.01) | Figure 5 and Table S5 |
| OL × CZ | F(3, 288)=4.46, P=0.00444 (P<0.01) | Figure 5 and Table S5 |
| SR × CZ | F(3, 288)=4.51, P=0.00413 (P<0.01) | Figure 5 and Table S5 |
| NC and/or IM × SB × CZ | F(9, 288)=0.90, P=0.529 | Figure 5 and Table S5 |
| NC and/or IM × VA × CZ | F(9, 288)=0.95, P=0.483 | Figure 5 and Table S5 |
| NC and/or IM × OL × CZ | F(9, 288)=0.88, P=0.543 | Figure 5 and Table S5 |
| NC and/or IM × SR × CZ | F(9, 288)=0.89, P=0.530 | Figure 5 and Table S5 |
| SB plus CZ × SR | F(1, 144)=3.95, P=0.0488 (P<0.05) | Figure 6 and Table S6 |
| VA plus CZ × SR | F(1, 144)=4.00, P=0.0474 (P<0.05) | Figure 6 and Table S6 |
| OL plus CZ × SR | F(1, 144)=4.08, P=0.0452 (P<0.05) | Figure 6 and Table S6 |
| NC and/or IM × SB plus CZ × SR | F(3, 144)=0.53, P=0.665 | Figure 6 and Table S6 |
| NC and/or IM × VA plus CZ × SR | F(3, 144)=0.52, P=0.672 | Figure 6 and Table S6 |
| NC and/or IM × OL plus CZ × SR | F(3, 144)=0.51, P=0.679 | Figure 6 and Table S6 |
